# Three new species of *Homatula* (Teleostei: Nemacheilidae) from Yunnan, China, with comments on habitat conservation

**DOI:** 10.1371/journal.pone.0276846

**Published:** 2022-11-23

**Authors:** Xu Li, Bo Yang, Yi Guo, Wei Zhou

**Affiliations:** 1 Key Laboratory for Conserving Wildlife with Small Populations in Yunnan, Southwest Forestry University, Kunming, Yunnan, China; 2 College of Biodiversity Conservation, Southwest Forestry University, Kunming, Yunnan, China; University of Wisconsin-Madison, UNITED STATES

## Abstract

Based on the morphological comparisons and molecular results, three new species of *Homatula*, i.e., *H*. *geminusclathratus* sp. nov., *H*. *microcephala* sp. nov., and *H*. *longibarbatus* sp. nov., have been described and named from the Lancang-jiang (the upper Mekong River) and the Chuan-he (the upper Black River, a tributary of the Red River) basins. The loaches of *Homatula* from the Lancang-jiang and the Chuan-he can be distinguished via morphology, genetics, and geographic distribution. All of the 10 recorded species distributed in the Nu-jiang (the upper Salween River), the Lancang-jiang, and the upper Black River share the following combination of character states: whole body, except head, densely scaled; lateral line complete; and a short adipose crest along the dorsal midline of the caudal peduncle, anteriorly not reaching vertically through the anal-fin origin. Species with these characters are called the densely-scaled group of *Homatula*. The three newly described species belong to the densely-scaled group of *Homatula*. Based on molecular phylogenetics, these *Homatula* species form a monophyletic group that can be divided into two clades, the densely-scaled group and the non-densely-scaled group. The densely-scaled group of *Homatula* includes 13 species occurring between the Nu-jiang and the upper Black River. The non-densely-scaled group is non-monophyletic and includes 14 species that are distributed in the Red, Pearl, Yangtze, and Yellow River basins. Species of the non-densely-scaled group are clustered into four sub-clades that are constrained to the four river basins. *Homatula* exclusively inhabits mountain streams with rapid or gentle currents, vauclusian springs, underground rivers connected to streams, and ditches near villages and farmland. No specimens of *Homatula* were collected from the main streams of Lixian-jiang, Lancang-jiang, and Nu-jiang as well as their large tributaries. Small environmental changes in the habitat of *Homatula*, such as water pollution or extensive human use, can lead to species/population extinction. Effective conservation of rare and endemic fishes, like loaches of *Homatula*, entails systematic observations and more targeted protection.

## 1 Introduction

The genus *Homatula* Nichols 1925 is a group of loaches that are endemic to China and presently known from the Yellow River, Yangtze River, Pearl River, Yuan-jiang (the upper reaches of the Red River), Lixiang-jiang (the upper reaches of the Black River, a tributary of the Red River), Lancan-jiang (the upper reaches of the Mekong River), and Nu-jiang (the upper reaches of the Salween River) basins [1–4]. Li, Che & Zhou [4] suggested that *Homatula* could be divided into two groups, the densely-scaled group and the non-densely-scaled group (i.e., the anterior body scaleless or the whole body scaleless) (Table 1). Ten species of *Homatula* are distributed in a narrow geographic area between the upper Salween River and the upper Black River (or Song Da in Vietnam, which is a tributary on the right bank of the Red River). Two species, *H*. *change* Endruweit (2015) and *H*. *coccinocola* Endruweit, Min & Yang (2018), are distributed in the upper Black River drainage [5, 6]; five species, *H*. *acuticephala* (Zhou & He, 1993), *H*. *anguillioides* (Zhu & Wang, 1985), *H*. *erhaiensis* (Zhu & Cao, 1988), *H*. *pycnolepis* Hu & Zhang (2010), and *H*. *wuliangensis* Min, Yang & Chen (2012), are distributed in the Lancang-jiang drainage [2, 7–10]; and three species, *H*. *anteridorsalis* Li, Che & Zhou (2019), *H*. *cryptoclathrata* Li, Che & Zhou (2019) and *H*. *nigra* Li, Che & Zhou (2019), are distributed in the Nu-jiang drainage [4].

**Table 1:** Comparison of characters between the densely-scaled and non-densely-scaled groups of *Homatula* (Li, Che & Zhou, 2019).

Group	Morphological characters		
	Scales on the body	Lateral line	Anterior of adipose crest along dorsal midline of caudal peduncle
Densely-scaled group	densely scaled body except head	complete	shorter adipose crest, anteriorly, not reaching vertically, through the anal-fin origin
Non-densely-scaled group	anterior body or entire body scaleless	complete or incomplete	longer adipose crest, anteriorly extending beyond the vertical through the anal-fin origin

However, the taxonomy of some *Homatula* species remains in question. Specifically, despite strong morphological evidence in support of the validity of both *H*. *acuticephala* and *H*. *erhaiensis* as two separate species [2, 4], molecular evidence indicates that these two species are not independent [6, 11]. However, these studies suffered from insufficient taxon and geographic range sampling, inadequate molecular evidence, and no use of an integrative taxonomic approach in their species delineation. Li, Che & Zhou [4] confirmed that the *H*. *pycnolepis* specimens recorded from Nu-jiang were misidentified as *H*. *pycnolepis* that holds a paired backwards-extending muscular protrusion between pelvic-fin bases, and corrected names have been proposed. In addition, while studying the loach specimens of *Homatula* from the Lancang-jiang and the upper reaches of Lixian-jiang, the authors found that the morphological characteristics of some *Homatula* specimens from different sites were significantly different from those of the recorded species.

Moreover, it was noted that *H*. *anguillioides*, *H*. *erhaiensis*, and *H*. *acuticephala* are limited to the area surrounding Erhai Lake, western Yunnan Province. In 2012, *H*. *anguillioides* and *H*. *acuticephala* were reported to be collected in the type localities [10]. *Homatula erhaiensis* has not been collected since the original report. In recent years, we have collected samples from the type localities of these three species, but no specimens have been collected. However, no additional populations of any species were found by extending the collection to areas outside the type locality of each species. *Homatula pycnolepis* Hu & Zhang 2010 is distributed in Yangbi-jiang, which belongs to the same small basin as Erhai Lake. Erhai Lake is connected to the Yangbi-jiang through the Xierh-he, which has dried up in sections due to the construction of small power stations. The habitat is no longer suitable for fishes and loaches to survive. Therefore, we are prompted to consider the survival of the *Homatula* loaches.

The aim of the present study was to clarify the taxonomy of *Homatula* species. Therefore, we conducted a systematic study of the *Homatula* species from the Lancang-jiang and the upper reaches of Lixian-jiang using both morphological taxonomy and molecular phylogenetics. Based on our extensive field investigations, we also suggested a conservation plan to ensure the protection of *Homatula* species.

## 2 Materials and methods

### 2.1 Ethics statement

All animal samples were obtained in compliance with the “Law of People’s Republic of China on the Protection of Wildlife” and “Regulations for the Implementation of the People’s Republic of China on the Protection of Aquatic Wildlife.” The care and use of experimental animals complied with animal welfare laws of the Chinese Laboratory Animal Welfare and Ethics. The scientific research ethics were reviewed and approved by the Academic Committee of Southwest Forestry University. All specimens were collected as part of routine surveys and killed humanely using approved procedures. When the individuals of live fish were collected in the field, they were firstly anesthetized with liquid prepared at ethyl 3-aminobenzoate methane sulphate (MSS 222). The anesthetic liquid was prepared and used as follows. The amount of MSS 222 was approximately 40–45 g/ m^3^ of water, and the ratio of fish to water was about 1:1. The fish were anesthetized after 15 to 20 minutes. Prior to fixation, a fin clip or muscle biopsy was taken and fixed separately in 95% ethanol solution. When returned to the laboratory, amples were then stored in anhydrous ethanol solution at -20°C until DNA extraction. The rest body of the fish was fixed and preserved in 95% ethanol or transferred to formalin solution.

### 2.2 Morphological measurements

All counts and measurements follow the methods presented in Kottelat [12]. For the anal and dorsal fins, ray counts are given in the following sequence: simple rays/branched rays; _1/2_ refers to last branched ray born by same pterygiophore as the penultimate ray. Caudal fin rays are indicated with the following notation: upper branched rays + lower branched rays [12]. Measurements were taken point to point with digital calipers and recorded to 0.1 mm. The methods presented in Zhu & Wang [7] were followed to count the bars on the flank; only those across the lateral line were counted. To facilitate the observations of the number of vertebra, X-ray photography was applied. X-rays were taken using a Digital Cabinet X-ray System (Xpeart 80, Kubtec, 270 Rowe Avenue, Unit E Milford, CT 06461, USA). The examined specimens of *Homatula* were from the Museum of Zoology, Southwest Forestry University, Kunming (SWFU) and the Kunming Institute of Zoology, Chinese Academy of Sciences, Kunming (KIZ). Abbreviations listed in the text and tables are: Co., county; ex. example; HL, head length; Pref., prefecture; Prov., province; SL, standard length. The suffixes -jiang and -he mean river and stream in Mandarin Chinese, respectively, and the suffix -hai means sea in Mandarin Chinese, but in Yunnan Province -hai means lake.

### 2.3 Study area and sample collection

The material used in the present study is the result of a collection of many field sampling trips throughout Yunnan Province conducted by several of the researchers over the past two decades. Specimens were collected using tools such as electrofishing, seine nets, cast nets, and gill nets across sites that encompass the diversity of running and still freshwater habitats in different basins in Yunnan. Specimens were individually labeled and voucher specimens were preserved in a 95% ethanol solution.

A total of 63 accessions were included in this study. The ingroup contained all 17 currently recognized species of *Homatula*, and the outgroup contained two species of *Barbatula*, two species of *Lefua*, two species of *Leptobotia*, two species of *Oreonectes*, two species of *Oxynoemacheilus*, one species of *Physoschistura*, three species of *Schistura*, one species of *Traccatichthys*, two species of *Triplophysa*, and one species of *Turcinoemachilus* (S1 Appendix). The specimens of *Homatula* were collected from the Nu-jiang, Lancang-jiang, and Lixiang-jiang drainage. Voucher specimens of the collected taxa were deposited at the Zoology Laboratory, Southwest Forestry University (SWFU). Additionally, sequences for other species of *Homatula* were obtained from GenBank [10, 13–15] (Table 2).

**Table 2:** Comparisons of characters among the species of the densely-scaled group of *Homatula* from the Nu-jiang (the upper Salween River), the Lancang-jiang (the upper Mekong River), and the upper Black River (a tributary of the Red River) in Yunnan Province, China.

Species	Type Locality (drainage)	Morphological Character States								
		Shape of lower jaw	A paired backwards muscular protrusions between pelvic-fin bases	Pattern of marks on the flank	Pelvic axillary lobe	Adipose crest anterior along dorsal midline of caudal peduncle	Location of anus between distance of pelvic-fin insertion and anal-fin origin	Terminal of maxillary barbel reach or extension	Gill opening size and its upper angle position	Length of dorsal-fin base/length of the longest branched dorsal-fin ray
*H*. *anteriordorsalis*	Bingmen Village, Longyang District, Yunnan (Nu-jiang)	V-shaped median notch	absent	dense barred pattern	present	*not reaching vertically through posterior end of anal-fin base*	located in the last 1/3	**almost reaching vertically below middle of the eye**	**smaller and its upper angle level with lower edge of the eye**	shorter
*H*. *cryptoclathratus*	Manping Village, Changning Co., Yunnan (Nu-jiang)	V-shaped median notch	absent	dense barred pattern	present	*not reaching vertically through posterior end of anal-fin base*	located in the last 1/3	**almost reaching vertically below middle of the eye**	**smaller and its upper angle level with lower edge of the eye**	**longer**
*H*. *niger*	Xiangshui Village, Changning Co., Yunnan (Nu-jiang)	V-shaped median notch	absent	*no dark pattern*	present	*not reaching vertically through posterior end of anal-fin base*	located in the last 1/3	**almost reaching vertically below middle of the eye**	**smaller and its upper angle level with lower edge of the eye**	shorter
*H*. *longibarbatus* sp. nov.	Pingpo Town, Yangbi Co., Yunnan (Lancang-jiang)	**spoon shaped and lacking a notch**	**present**	dense barred pattern	present	not extending beyond vertically through middle of anal-fin base	**located in the last 1/4**	extending beyond vertically below posterior edge of the eye	larger and its upper angle aligned with middle point of the eye	shorter
*H*. *microcephala* sp. nov.	Shili-He, Yunlong Co., Yunnan (Lancang-jiang)	**spoon shaped and lacking a notch**	**present**	dense barred pattern	present	not extending beyond vertically through middle of anal-fin base	located in the last 1/3	**almost reaching vertically below posterior edge of the eye**	**smaller and its upper angle level with lower edge of the eye**	shorter
*H*. *geminusclathratus* sp. nov.	Zhenyuan Co., Yunnan (Lancang-jiang)	**spoon shaped and lacking a notch**	absent	dense barred pattern	present	**reaching vertically through anal-fin origin**	**located in the last 1/4**	**almost reaching vertically below posterior edge of the eye**	larger and its upper angle aligned with middle point of the eye	**longer**
*H*. *wuliangensis*	Jingfu Town, Jingdong Co., Yunnan (Lancang-jiang)	**spoon shaped and lacking a notch**	absent	dense barred pattern	present	not extending beyond vertically through middle of anal-fin base	**located in the last 1/4**	**almost reaching vertically below middle of the eye**	**smaller and its upper angle level with lower edge of the eye**	**longer**
*H*. *erhaiensis*^§^	Eastern shore of Er-Hai Lake, Yunnan (Lancang-jiang)	**spoon shaped and lacking a notch**	absent	**sparse barred pattern**	present	not extending beyond vertically through middle of anal-fin base	located in the last 1/3	**almost reaching vertically below posterior edge of the eye**	larger and its upper angle aligned with middle point of the eye	**longer**
*H*. *pycnolepis*	Shaxi Town, Jianchuan Co., Yunnan (Lancang-jiang)	V-shaped median notch on lower jaw	**present**	dense barred pattern	present	not extending beyond vertically through middle of anal-fin base	located in the last 1/3	**almost reaching vertically below posterior edge of the eye**	larger and its upper angle aligned with middle point of the eye	shorter
*H*. *acuticephala*	Haixi-Hai, Eryuan Co., Yunnan (Lancang-jiang)	V-shaped median notch on lower jaw	absent	**sparse barred pattern**	**absent**	not extending beyond vertically through middle of anal-fin base	located in the last 1/3	**almost reaching vertically below middle of the eye**	larger and its upper angle aligned with middle point of the eye	**longer**
*H*. *anguillioides*	Yousuo Spring, Eryuan Co., Yunnan (Lancang-jiang)	V-shaped median notch	absent	dense barred pattern	**absent**	not extending beyond vertically through middle of anal-fin base	located in the last 1/3	extending beyond vertically below posterior edge of the eye	larger and its upper angle aligned with middle point of the eye	**longer**
*H*. *change*	Yiwanshui Village, Jiangcheng Co., Yunnan (upper Black River)	V-shaped median notch	absent	dense barred pattern	present	*not reaching vertically through posterior end of anal-fin base*	located in the last 1/3	extending beyond vertically below posterior edge of the eye	**smaller and its upper angle level with lower edge of the eye**	shorter
*H*. *coccinocola*	Jiache Town, Honghe Co., Yunnan (upper Black River)	V-shaped median notch	absent	**sparse barred pattern**	present	not extending beyond vertically through middle of anal-fin base	located in the last 1/3	*reaching vertically below posterior edge of the eye*	larger and its upper angle aligned with middle point of the eye	shorter

^§^ Data from Zhu & Cao (1988). In each column, the same font formatting (bold or italic) indicates that different species have the same morphological character state.

### 2.4 Nomenclatural acts

The electronic edition of this article conforms to the requirements of the amended International Code of Zoological Nomenclature, and hence the new names contained herein are available under that Code from the electronic edition of this article. This published work and the nomenclatural acts it contains have been registered in ZooBank, the online registration system for the ICZN. The ZooBank LSIDs (Life Science Identifiers) can be resolved and the associated information viewed through any standard web browser by appending the LSID to the prefix "http://zoobank.org/". The LSID for this publication is: urn:lsid:zoobank.org:pub:B95E1DE2-E2C2-4A3E-BE7F-754704AD6F1E. The electronic edition of this work was published in PLoS One with an ISSN 1932-6203.

### 2.5 Molecular methods

#### 2.5.1 DNA extraction and PCR

DNA was extracted from the ethanol-preserved fin clips using a standard phenol-chloroform extraction method [16]. The complete cytochrome *b* (Cyt *b*) gene sequence was amplified using the primer pair L14724 (5′-GAC TTG AAA AAC CAC CGT TG-3′) and H15915 (5′-CTC CGA TCT CCG GAT TAC AAG AC-3′) [17].

Polymerase chain reactions (PCR) were performed in a total volume of 25 μL consisting of 9.5 μL dd H_2_O, 1.0 μL DNA template, 1.0 μL each primer (10 μM), and 12.5 μL 2×Taq PCR Master Mix (TIANGEN; Beijing, China). The PCR conditions used were as follows: an initial denaturation step at 94°C for 5 min; 35 cycles of 30 s at 94°C, 45 s at 53°C, and 50s at 72°C; and a final extension for 10 min at 72°C.

#### 2.5.2 Molecular data analysis

DAMBE 5.3.48 was used to test nucleotide substitution saturation [18]. Alignment of the Cyt *b* sequences was performed using the Clustal W algorithm implemented in MEGA 7 [19], with subsequent manual checks for inconsistencies and stop codons. The aligned sequences were analyzed using the Bayesian inference (BI) method in MrBayes 3.2.6 [20]. The nucleotide substitution model was determined with ModelTest 3.7, and had the following results: Base = (0.5124, 1.5451, 1.0000), Nst = 6, Rmat = (0.2144, 0.2646, 0.1749, 0.3462, 1.0000), Rates = equal. The Generalized Time Reversible, equal-distributed rate variation, and the proportion of invariable positions (GTR+G+I) for the Cyt *b* datasets were used. Markov Chain Monte Carlo (MCMC) simulations were run for 2,000,000 generations, with sampling every 1,000 generations, and the first 25% of samples were discarded as burn-in. The resulting phylogeny was visualized and edited in FigTree v. 1.4.2 [21].

## 3 Results

Our morphological and molecular results confirmed that *Homatula geminusclathratus* sp. nov., *H*. *longibarbatus* sp. nov., and *H*. *microcephala* sp. nov. are valid species.

### 3.1 Morphological comparisons among species of *Homatula*

Systematic comparison of the specimens from the Nu-jiang, Lancang-jiang, and Lixiang-jiang drainages indicates that there are 13 valid species, including three undescribed new species. All 13 species share the following combination of character states: densely scaled body except head; lateral line complete; and a short adipose crest along the dorsal midline of the caudal peduncle, situated anteriorly not reaching vertically through the anal-fin origin (Tables 1 and 2). Thus, these species belong to the densely-scaled group of the genus *Homatula*.

#### 3.1.1 *Homatula geminusclathratus* sp. nov. (Figs 1, 2A and 3A)

**Fig 1:**
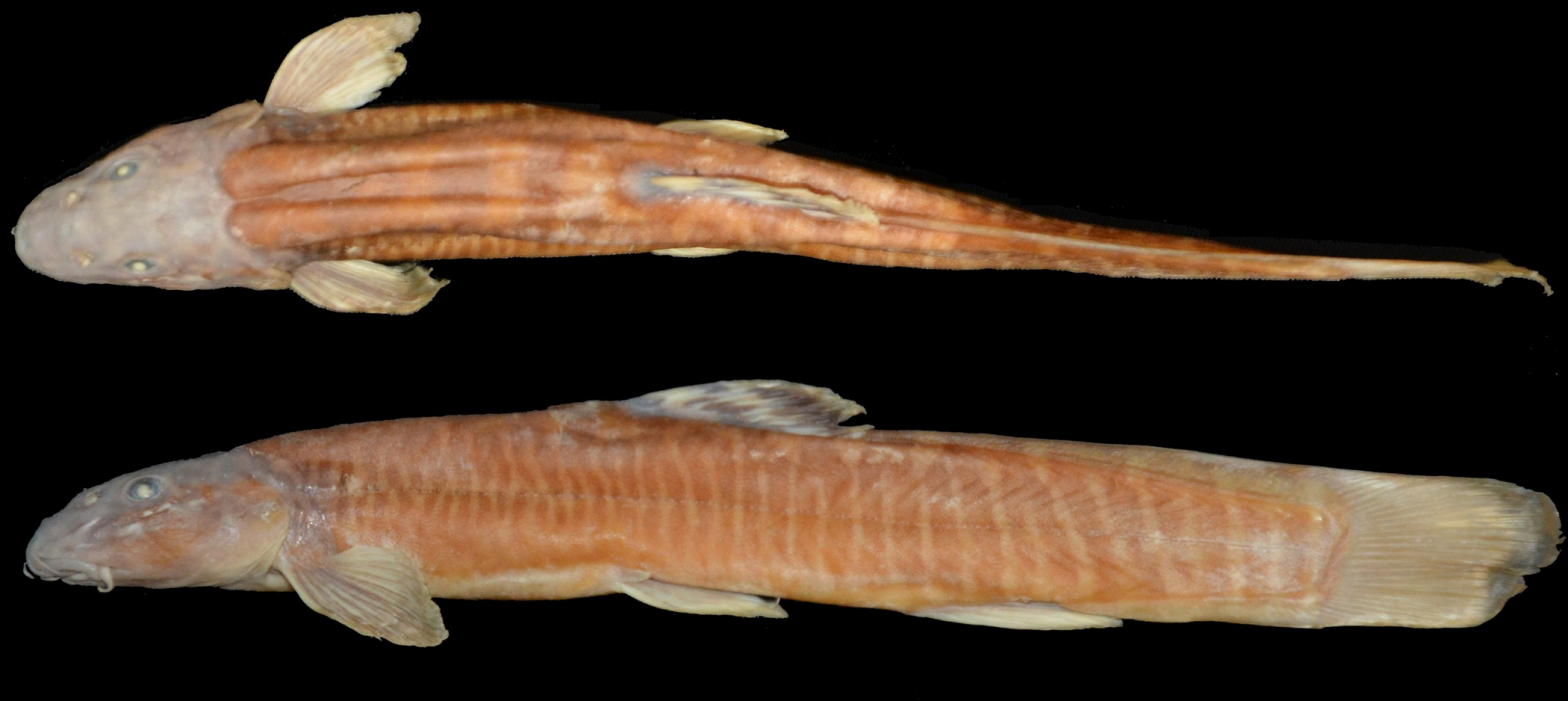
Left lateral and dorsal views of *Homatula geminusclathratus*. Holotype SWFU 0309250, 131.8 mm SL; China: Yunnan Prov.: Pu’er City: Jingdong Co.: Wenjing Town.

**Fig 2:**
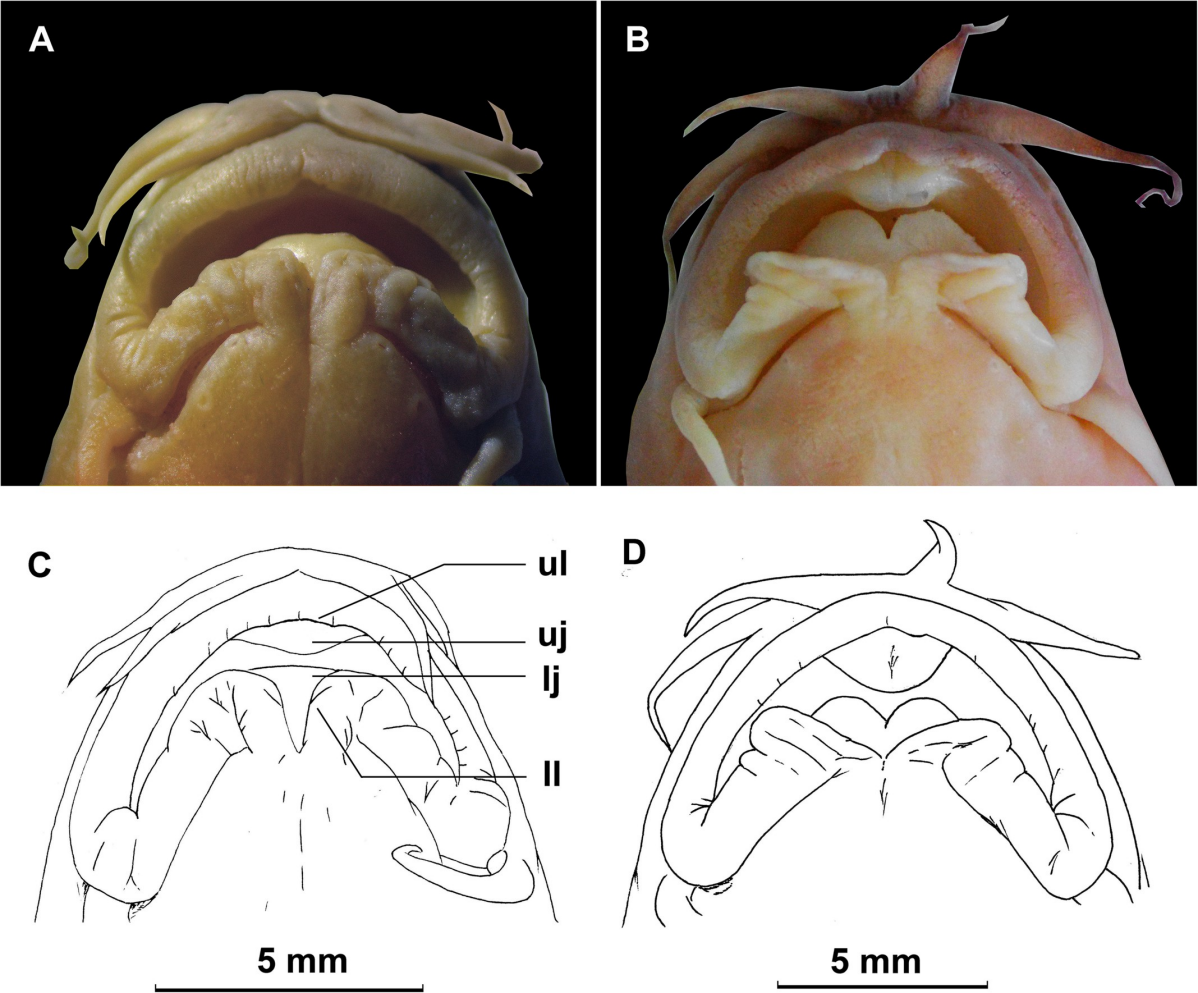
Ventral view of the mouth and shape of the lower jaw. (A, C) Spoon shaped and lacking a median notch, *H*. *geminuclathratus*, Paratype SWFU 0309054, 113.4 mm SL; China: Yunnan Prov.: Pu’er City: Jingdong Co.: Wenjing Town. (B, D) Shallow V-shaped median notch on lower jaw, *H*. *change*. SWFU 0412001, 115.3 mm SL; China: Yunnan Prov.: Pu’er City: Jiangcheng Co.: Jima-he. (ul: upper lip; ll: lower lip; uj: upper jaw; lj: lower jaw).

**Fig 3:**
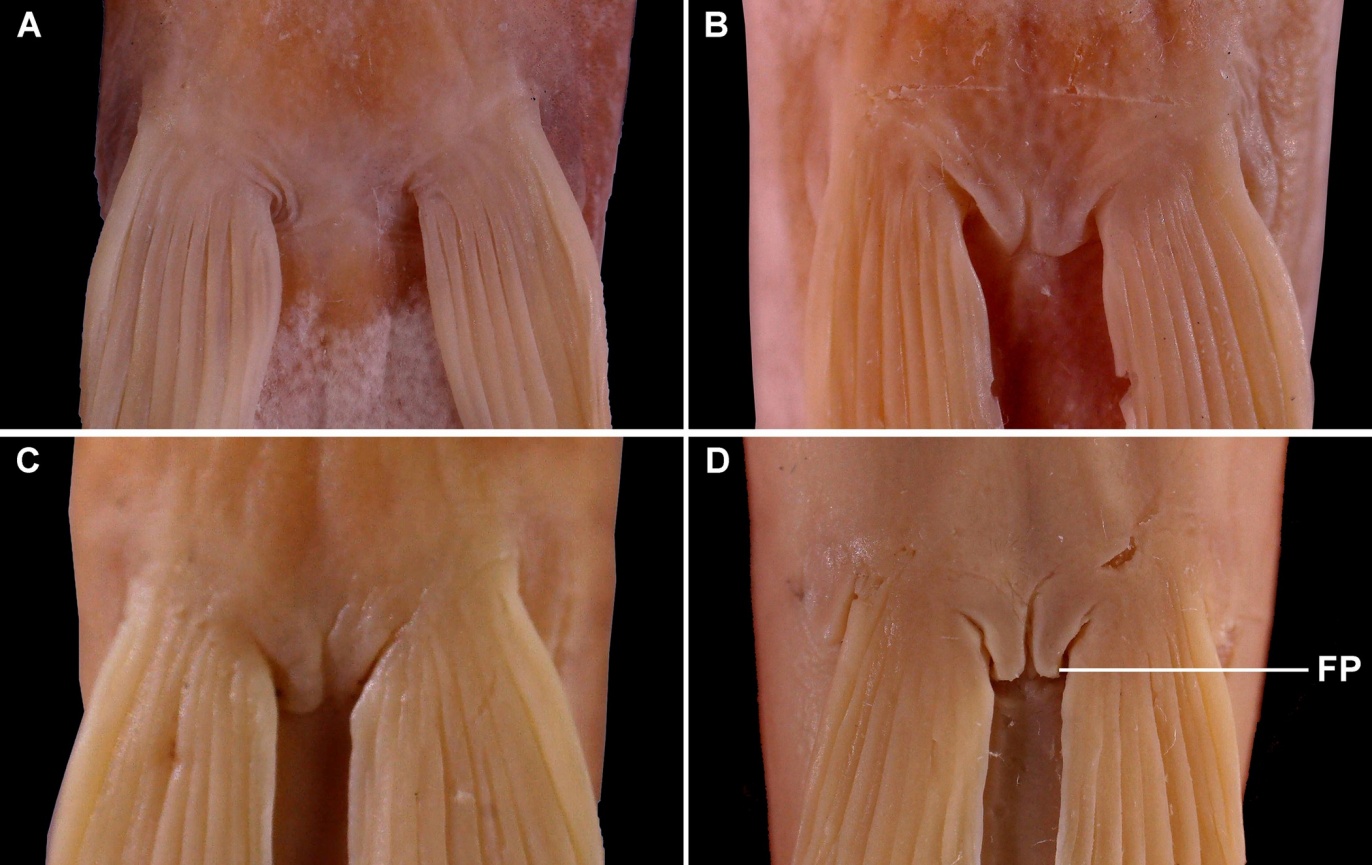
Ventral view of pelvic plate of *Homatula* from the upper Mekong River, China. (A) *H*. *geminusclathratus*, holotype SWFU 0309250, 131.8 mm SL; China: Yunnan Prov.: Pu’er City: Jingdong Co.: Wenjing Town. (B) *H*. *longibarbatus*, holotype SWFU 0309082, 107.3 mm SL; China: Yunnan Prov.: Dali Pref.: Yangbi Co.: Pingpo Town. (C) *H*. *microcephala*, holotype SWFU 0612057, 90.2 *mm* SL; China: Yunnan Prov.: Dali Pref.: Yunlong Co.: Jiancao Town. (D) *H*. *pycnolepis*, SWFU 0612085, 99.6 mm SL; China: Yunnan Prov.: Dali Pref.: Yunlong Co.: Xiangtu Town. (FP: A muscular protrusion in body surface at the pelvic plate).

LSID: zoobank.org: act: 028C9327-D49E-4016-8804-5F285EBD4CBA

**Holotype.** SWFU 0309250, 131.8 mm SL; China: Yunnan Prov., Pu’er City, Jingdong Co., Wenjing Town, Xiaobahe ([not a river, just the name of a location] on the eastern slope of Wuliangshan), Chuan-he (24°19’53.97" N, 100°48’37.17" E); collected by Q. Wang & W. Zhou, 29 Sep. 2003.

**Paratypes.** SWFU 0309251–0309272, 22 ex., 49.5–132.4 mm SL; the other data are the same as the holotype.

**Fig 4:**
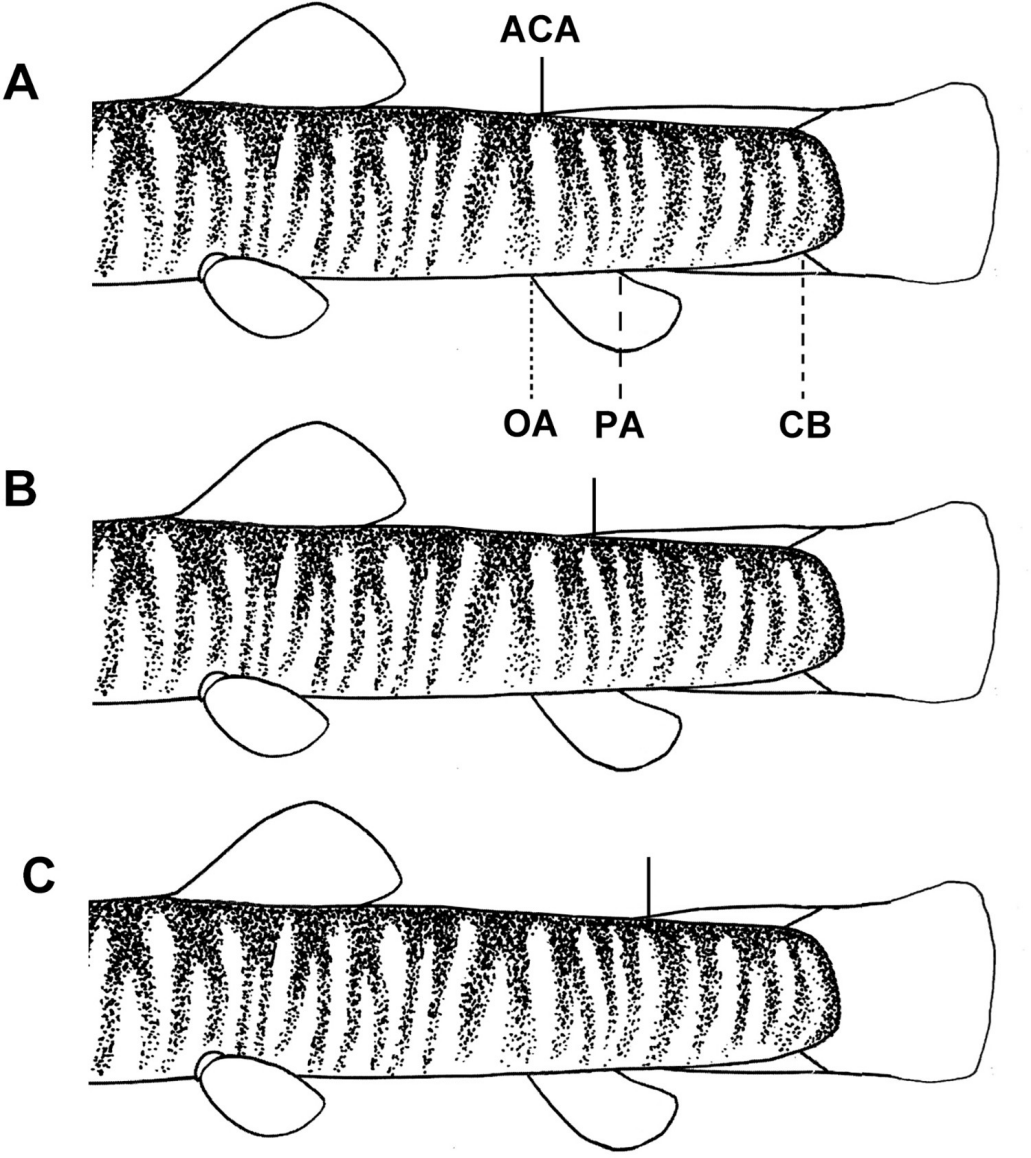
Location of the adipose-crest anterior along dorsal midline of caudal peduncle in *Homatula*. (A) Anteriorly reaching vertically through anal-fin origin; (B) Anteriorly not extending beyond vertical through the middle of the anal-fin base; (C) Anteriorly not reaching vertically through the posterior end of the anal-fin base. ACA: adipose crest anterior; CB: caudal-fin base; OA: origin of anal fin; PA: posterior of anal-fin base.

**Diagnosis.** *Homatula geminusclathratus* differs from congeners with a densely-scaled body by the following combination of character states: lower jaw spoon-shaped without a median notch (Fig 2A and 2C) (*v*. with a marked V-shaped median notch in *H*. *acuticephala*, *H*. *anguillioides*, *H*. *anteriordorsalis*, *H*. *change*, *H*. *coccinocola*, *H*. *cryptoclathratus*, *H*. *niger*, and *H*. *pycnolepis*) (Fig 2B and 2D); no paired backwards-extended muscular protrusions between pelvic-fin bases (Fig 3A) (*v*. with paired backwards-extended muscular protrusions between pelvic-fin bases in *H*. *longibarbatus* sp. nov., *H*. *microcephala* sp. nov., and *H*. *pycnolepis*) (Fig 3B–3D); bars on the flank across the lateral line no fewer than 25 (*v*. no more than 20 in *H*. *acuticephala*, *H*. *coccinocola*, and *H*. *erhaiensis*); adipose crest along mid-dorsal line of caudal peduncle anteriorly reaching vertically through the anal-fin origin (Fig 4A) (*v*. not vertical through posterior end of anal-fin base in *H*. *anteriordorsalis*, *H*. *change*, *H*. *cryptoclathratus*, and *H*. *niger*; or not extending beyond vertical through middle of anal-fin base in *H*. *acuticephala*, *H*. *anguillioides*, *H*. *coccinocola*, *H*. *erhaiensis*, *H*. *longibarbatus* sp. nov., *H*. *microcephala* sp. nov., *H*. *pycnolepis*, and *H*. *wuliangensis*) (Fig 4B and 4C) (Tables 2 and 3).

**Table 3:** Morphometric data for three new species of *Homatula* from the Lancang-jiang, Yunnan Province, China.

	*Homatula geminusclathratus* sp. nov.					*Homatula posteriordorsal* sp. nov.					*Homatula microcephala* sp. nov.				
	Holotype	Paratypes (22)				Holotype	Paratypes (30)				Holotype	Paratypes (30)			
		Min	Max	Mean	SD		Min	Max	Mean	SD		Min	Max	Mean	SD
Standard length (mm)	131.8	49.5	132.4	87.4	24.8	107.3	73.0	117.7	93.8	11.8	90.2	51.6	130.6	89.7	23.7
Head length (mm)	27.9	10.6	27.9	18.5	5.1	21.6	15.8	24.7	20.6	2.2	18.6	11.5	26.9	18.7	4.4
Head width (mm)	16.5	6.1	17.6	11.1	3.2	14.3	9.9	15.2	12.5	1.4	10.4	6.0	16.0	10.0	2.5
Head depth (mm)	13.5	5.2	13.7	9.2	2.5	11.2	8.0	12.5	10.4	1.1	8.9	5.1	12.1	8.2	1.9
**% of standard length**															
Body depth (at dorsal-fin origin)	14.4	13.0	15.1	14.2	0.6	14.0	13.3	15.8	14.7	0.7	13.5	13.0	15.6	14.0	0.6
Body depth (at anus)	13.3	12.3	14.9	13.1	0.7	12.3	11.9	13.8	12.9	0.5	12.4	11.3	12.9	12.3	0.5
Head length	21.2	20.0	22.6	21.2	0.6	20.1	20.6	23.3	22.0	0.8	20.6	20.2	22.4	21.1	0.7
Head width	12.5	11.4	13.5	12.8	0.5	13.3	11.4	14.9	13.4	0.9	11.5	10.0	12.9	11.3	0.7
Head depth	10.2	9.5	11.3	10.5	0.5	10.4	10.0	12.4	11.1	0.6	9.9	8.2	10.0	9.3	0.5
Length of caudal peduncle	21.8	19.4	21.8	20.6	0.7	18.9	18.3	20.7	19.3	0.8	18.6	18.1	20.7	19.2	0.6
Depth of caudal peduncle	7.4	6.1	8.2	7.1	0.6	6.4	6.1	7.5	6.8	0.4	7.5	6.3	8.8	7.3	0.7
Pectoral-fin length	15.4	15.3	18.0	16.1	0.8	14.8	15.3	18.0	16.7	0.8	15.1	14.3	16.0	15.4	0.5
Anal-fin length	14.8	14.4	16.7	15.5	0.7	15.2	13.5	17.6	16.0	0.9	16.5	14.0	16.2	14.8	0.5
Caudal-fin length	17.4	17.5	19.7	18.3	0.7	16.0	14.8	17.1	16.3	0.5	15.0	14.0	16.7	15.0	0.7
Dorsal-fin length	19.6	18.5	20.9	19.9	0.7	20.4	16.9	21.6	19.7	1.0	18.9	18.2	21.0	19.8	0.8
Pelvic-fin length	13.6	13.2	16.0	14.5	0.8	13.1	11.4	15.5	13.9	0.9	12.3	11.0	13.8	12.4	0.8
Predorsal length	47.4	46.4	49.0	47.8	0.7	47.5	48.7	52.1	50.1	0.8	48.0	47.2	49.7	48.3	0.7
Prepelvic length	47.7	46.9	48.8	47.7	0.4	51.1	49.5	52.8	51.4	0.8	50.0	48.1	50.9	49.9	0.7
Prepectoral length	19.7	19.2	21.9	20.7	0.7	21.3	20.4	23.5	22.2	0.8	21.2	20.0	22.7	21.3	0.9
Preanal length	71.4	70.0	73.0	71.3	0.9	72.3	70.6	73.2	72.1	0.6	73.6	71.0	73.8	72.5	0.8
Preanus length	66.9	66.1	68.8	67.1	0.7	67.2	64.4	68.5	67.0	0.9	66.9	65.4	67.9	66.7	0.7
**% of length of caudal peduncle**															
Depth of caudal peduncle	34.2	29.8	39.3	34.4	2.8	33.7	30.8	39.9	35.4	2.2	40.5	32.6	46.2	38.3	3.4
**% of head length**															
Snout length	39.5	39.0	42.2	40.6	1.0	39.6	37.9	40.9	39.4	0.8	47.3	41.5	44.7	43.4	0.9
Eye diameter	17.0	14.6	18.4	16.6	1.1	15.1	12.3	15.1	13.9	0.7	13.6	13.1	15.7	14.2	0.8
Interorbital width	27.4	26.1	30.1	28.4	1.0	28.8	24.3	27.8	26.5	0.8	22.0	21.5	25.2	23.2	1.0

**Description.** Based on the holotype and 22 paratypes, the maximum SL is 132.4 mm. Morphometric data are shown in Table 3. Dorsal-fin rays iii, 8_1/2_; anal-fin rays iii, 5_1/2_; pectoral-fin rays i, 10–11; pelvic-fin rays i, 7; caudal-fin branched rays 9+8; vertebra 4+42 (n = 3).

Body elongate and cylindrical, anteriorly slightly depressed and posteriorly compressed laterally. Maximum body depth immediately in front of dorsal fin. Dorsal and ventral profiles almost straight. Excepting the head and thorax, body entirely covered by small scales, with scattered small scales on the abdomen. Caudal peduncle compressed laterally. Lateral line complete, extending directly along midline of body.

Head short and depressed in frontal view, wider than high, roughly triangular in dorsal view. Snout blunt, slightly shorter than postorbital head. Eye small, close to dorsal profile of head, not visible from ventral view. Interorbital space wide and flat. Nostrils closely set, nearer to anterior margin of eye than to snout tip; anterior nostrils situated at a nostril valve.

Mouth inferior and arched (Fig 2A and 2C). Lips thick, slightly furrowed, but not papillated; upper lip with a small median incision, and lower lip with a marked median incision. Jaws covered by lips; upper jaw with a well-developed *processus dentiformis*, lower jaw spoon shaped and lacking a notch (Fig 2C). Three pairs of barbels: two rostral pairs, inner pair not reaching corners of mouth, outer pair reaching a vertical line of anterior nostril; one short maxillary pair, reaching vertically almost to posterior margin of the eye.

Dorsal fin distal margin convex as a whole; dorsal and pelvic origins slightly forward, midway between snout tip to caudal-fin base. Pectoral fin inserted slightly posterior to vertical through posteriormost point of operculum, tip of adpressed fin rays reaching halfway to insertion of pelvic fin. Pelvic fin inserted below first or second branched rays of dorsal fin, tip of adpressed fin ray approaching half the distance between pelvic-fin insertion and anal-fin origin. Pelvic axillary lobe with pointed tip. Anal fin with convex distal edge; origin closer to pelvic-fin insertion than to caudal-fin base. Posterior margin of caudal fin oblique or micro-concave. Caudal peduncle uniformly deep, with adipose crests along its dorsal and ventral midlines. Adipose crest along dorsal midline of caudal peduncle anteriorly extending vertically through the anal-fin origin (Fig 4A). Anus located approximately posterior, 1/4 distance between the pelvic-fin insertion and anal-fin origin, closer to the caudal-fin base.

**Coloration:** The flank orange yellow with 36 to 38 vertical brown bars along body lateral line and the abdomen grayish white. The flank with 36 to 38 vertical brown bars along body lateral middle axisline. Width of bars larger than the interspace. Adjacent stripes connected on the dorsum of the body and regularly arranged in pairs. Dark brown vertical bar on caudal-fin base. Dorsal fin orange-yellow, two rows of brown spots on dorsal fin. Pectoral, pelvic, and anal fins orange-yellow, distal margin of each fin orange-red, and fin rays brown. Caudal fin yellowish, its distal margin orange-red. Dorsal and ventral adipose crest orange-yellow and outer margin of the upper adipose orange-red. After fixation in 95% alcohol, the body color changed from orange-yellow to brown, brown stripes and spots faded, and distal margin edge of each fin orange-red disappeared to gray.

**Fig 5:**
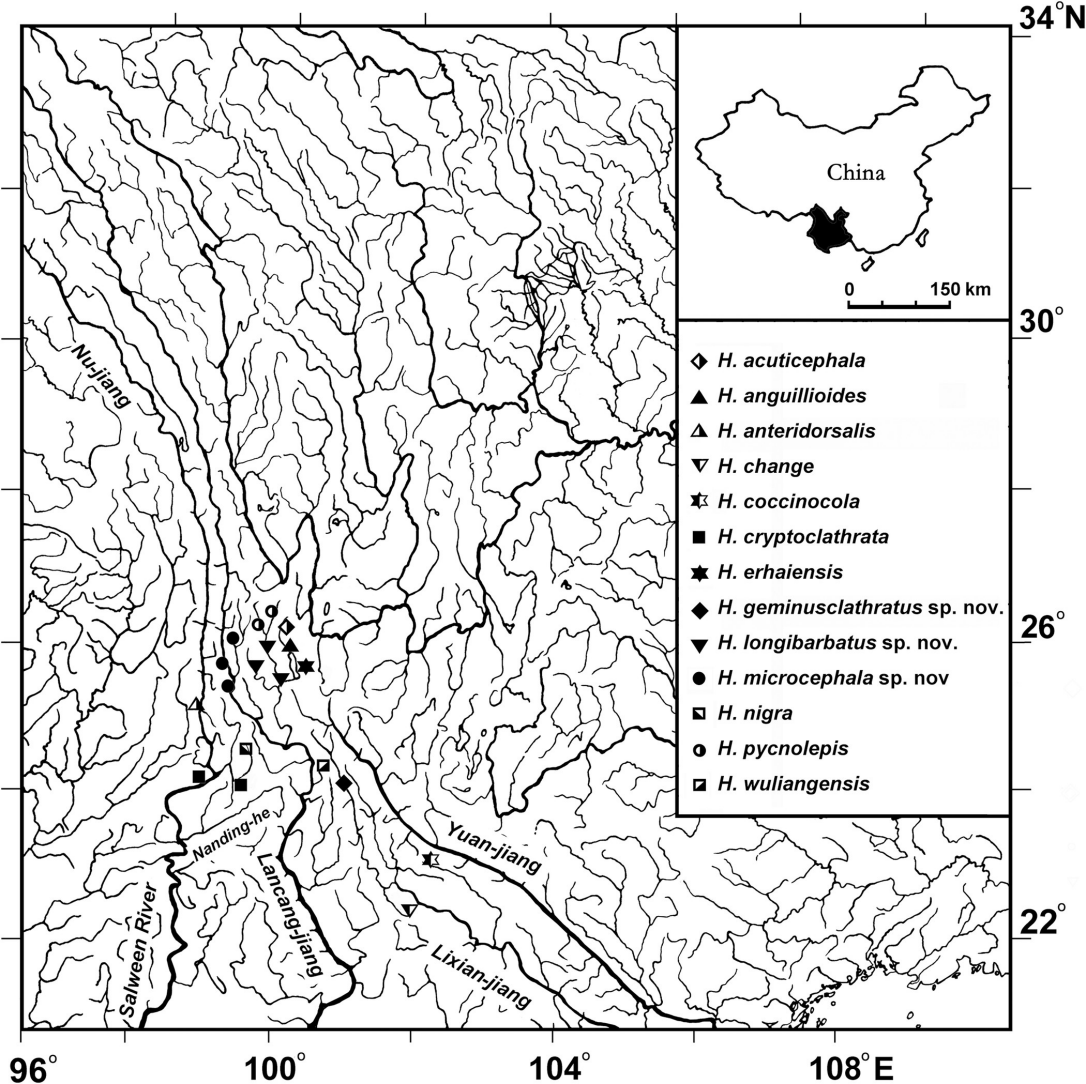
Distribution map of the densely-scaled group of *Homatula*. A symbol may represent several adjacent localities.

**Distribution:** Known from the Chuan-he in Jingdong Co., the upper Black River (Fig 5).

**Habitat and Ecology:** *Homatula geminusclathratus* lives in streams with a rock matrix in valleys. The bottom of the stream was strewn with sand and gravel, and the current was swift. *Homatula geminusclathratus* is omnivorous, mainly feeding on algae attached to rocks, organic residue, and small aquatic insects. It usually coexists with *Schistura* spp. (Nemacheilidae) and *Pareuchiloglanis* spp. (Sisoridae).

**Etymology:** From the Latin *geminus*–(paired) and–*clathratus* (barred), alluding to the regularly arranged, paired bars on the flanks. Used as an adjective.

#### 3.1.2 *Homatula longibarbatus* sp. nov. (Figs 2B, 3B and 6)

**Fig 6:**
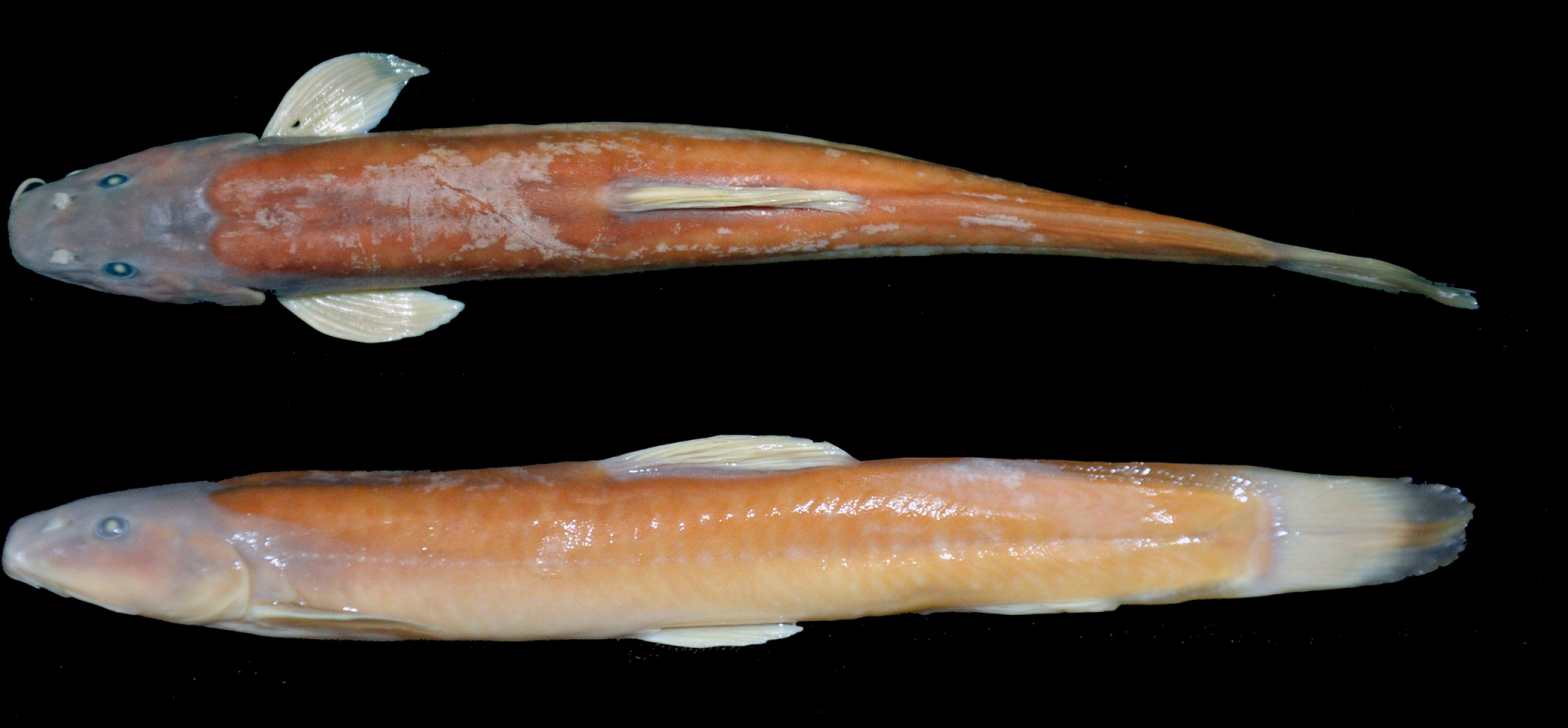
Left lateral and dorsal views of *Homatula longibarbatus*, holotype SWFU 0309082, 107.3 mm SL; China: Yunnan Prov.: Dali Pref.: Yangbi Co.: Pingpo Town.

LSID: zoobank.org: act: 28DC57EB-E2B5-4E56-AFB5-C0E6F5E3D032

*Homatula pycnolepis*: Min et al. 2012a: 81 (Yunnan: Yangbi: Lancang-jiang).

**Holotype.** SWFU 0309082, 107.3 mm SL; China: Yunnan Prov.: Dali Pref.: Yangbi Co.: Pingpo Town (25°35’53.36"N, 100°3’36.10"E); collected by Y. Yang & J.F. He, 8 Sep. 2003.

**Paratypes.** SWFU 0309078–03081, 0309083–0309089, 11 ex., 90.0–117.7 mm SL; the other data are the same as the holotype; SWFU 0309090–0309108, 19 ex., 73.0–94.2 mm SL; China: Yunnan Prov.: Dali Pref.: Eryuan Co.: Qiaohou Town (26°5’53.91"N, 99°47’9.97"E); collected by Y. Yang & J.F. He, 10 Sep. 2003. SWFU 0309131–0309136, 6 ex., 87.6–117.3 mm SL; China: Yunnan Prov.: Dali Pref.: Yangbi Co.: Yuejin Town (25°29’7.41"N, 99°55’36.06"E); collected by Y. Yang & J.F. He, 10 Sep. 2003.

**Other examined specimens.** KIZ 2009005388, 1 ex., 141.2 mm SL; China: Yunnan Prov.: Dali Pref.: Yangbi Co.: Wachang Town; KIZ 1998004817, 1998004819, 1998004822, 1998004827, 4 ex., 132.4–178.6 mm SL; China: Yunnan Prov.: Dali Pref.: Yangbi Co.; KIZ 2010002517–18, 2010002521–24, 2010002532, 2010002536, 2010002549, 9 ex., 103.7–137.5 mm SL; China: Yunnan Prov.: Dali Pref.: Yangbi Co.; KIZ 2009003860–62, 2009003865, 2009003869, 5 ex., 64.5–97.4 mm SL; China: Yunnan Prov.: Dali Pref.: Yunlong Co.: Tuanjie Town: Guanping-he.

**Diagnosis.** *Homatula longibarbatus* differs from congeners with a densely-scaled body by the following combination of character states: lower jaw spoon-shaped and without a median notch (Fig 2C) (*v*. with a marked V-shaped median notch in *H*. *acuticephala*, *H*. *anguillioides*, *H*. *anteriordorsalis*, *H*. *change*, *H*. *coccinocola*, *H*. *cryptoclathratus*, *H*. *niger*, and *H*. *pycnolepis*) (Fig 2B and 2D); paired backwards-extended muscular protrusions between pelvic-fin bases (Fig 3B) (*v*. without paired backwards-extended muscular protrusions between pelvic-fin bases in *H*. *acuticephala*, *H*. *anguillioides*, *H*. *anteriordorsalis*, *H*. *change*, *H*. *coccinocola*, *H*. *cryptoclathratus*, *H*. *erhaiensis*, *H*. *geminusclathratus* sp. nov., *H*. *niger*, and *H*. *wuliangensis*) (Fig 3A); bars on flank across the lateral line no fewer than 25 (*v*. no more than 20 in *H*. *acuticephala*, *H*. *coccinocola*, and *H*. *erhaiensis*); maxillary barbel long, extending beyond vertically through posterior of the eye (*v*. maxillary barbel short, not reaching vertically beyond the posterior of the eye in *H*. *acuticephala*, *H*. *anteriordorsalis*, *H*. *coccinocola*, *H*. *cryptoclathratus*, *H*. *erhaiensis*, *H*. *geminusclathratus* sp. nov., *H*. *microcephala* sp. nov., *H*. *niger*, *H*. *pycnolepi*, and *H*. *wuliangensis*) (Tables 2 and 3).

**Description.** Based on the holotype and 30 paratypes, the maximum SL is 117.7 mm. Morphometric data are shown in Table 3. Dorsal-fin rays iii, 8_1/2_; anal-fin rays iii, 5_1/2_; pectoral-fin rays i, 9–10; pelvic-fin rays i, 7; caudal-fin branched rays 9+8; vertebra 4+41 (n = 3).

Body elongate and cylindrical, anteriorly slightly depressed and posteriorly compressed laterally. Maximum body depth immediately in front of dorsal fin. Dorsal and ventral profiles almost straight. Excepting the head and thorax, body entirely covered by small scales, with scattered small scales on the abdomen. Caudal peduncle compressed laterally. Lateral line complete, extending directly along midline of body.

Head short and depressed in frontal view, wider than high, roughly triangular in dorsal view. Snout blunt, slightly shorter than postorbital head. Eye small, close to dorsal profile of head, invisible from ventral view. Interorbital space wide and flat. Nostrils closely set, nearer to anterior margin of eye than to snout tip; anterior nostrils situated at a nostril valve.

Mouth inferior and arched (Fig 2C). Lips thick, slightly furrowed, but not papillated; upper lip with a small median incision, and lower lip with a marked median incision. Jaws covered by lips; upper jaw with a well-developed *processus dentiformis*. Lower jaw spoon shaped and lacking a notch (Fig 2C). Three pairs of barbels: two rostral pairs, inner pair not reaching corners of mouth, outer pair reaching a vertical line of anterior nostril, and maxillary pair long, extending beyond vertical through posterior margin of the eye.

Dorsal fin distal margin convex as a whole; dorsal and pelvic origins slightly before midway between snout tip and to caudal-fin base. Pectoral fin inserted slightly posterior to vertical through posteriormost point of operculum; tip of adpressed fin rays reaching halfway to insertion of pelvic fin. Pelvic fin inserted below first or second branched rays of dorsal fin; tip of adpressed fin ray approaching halfway between pelvic-fin insertion and anal-fin origin. Pelvic axillary lobe with pointed tip. Anal fin with convex distal edge; origin closer to pelvic-fin insertion than to caudal-fin base. Posterior margin of caudal fin oblique or micro-concave. Caudal peduncle uniformly deep, with adipose crests along dorsal and ventral midlines of caudal peduncle. Adipose crest along dorsal midline of anterior caudal peduncle, almost vertically extending to the middle of the anal-fin base (Fig 4A). Anus located approximately posterior, 1/4 distance between the pelvic-fin insertion and anal-fin origin, closer to the caudal-fin base.

**Coloration:** When alive, the flank brownish, dark brown on the back, and the abdomen grayish yellow. The flank with 25 to 29 vertical chocolate brown bars along lateral middle axis of the body. Some of bars joined and forming saddles on the back. Width of bars approximately equal to or narrower than the interspace. The bars in front of anal-fin origin fine and dense, gradually widening to the caudal-fin base. Dark brown vertical bar on caudal-fin base. Dorsal, pectoral, pelvic, and anal fins reddish orange, distal margin of each fin orange-red, and fin rays brown. Caudal fins pale yellow, its distal margin orange-red. Dorsal and ventral adipose crest orange-yellow. After fixation in 95% alcohol, brownish yellow body color and chocolate brown bars faded, and orange-red distal edge of each fin disappeared to gray.

**Distribution:** Known only from the Heihui-jiang, which is a first-class tributary of the Lancang-jiang (upper Mekong River drainage) (Fig 5).

**Habitat and Ecology:** *Homatula longibarbatus* inhabits flowing and clear stream environments, dwelling on the bottom, shuttling back and forth among rocks and small stones. *Homatula longibarbatus* is omnivorous, mainly feeding on algae attached to rocks, organic detritus, and small aquatic insects. It usually coexists with *Homaloptera* spp. (Homalopteridae), *Schistura* spp. (Nemacheilidae), *Vanmanenia* spp. (Balitoridae), and *Glyptothorax* spp. (Sisoridae).

**Etymology:** The specific epithet is a combination of the Latin words *long*–(long) and–*barbatus* (barbel), indicating its long maxillary barbel, which extends beyond a vertical line at the posterior margin of the eye. The Latin words are used as adjectives.

#### 3.1.3 *Homatula microcephala* sp. nov. (Figs 2C, 3C and 7)

**Fig 7:**
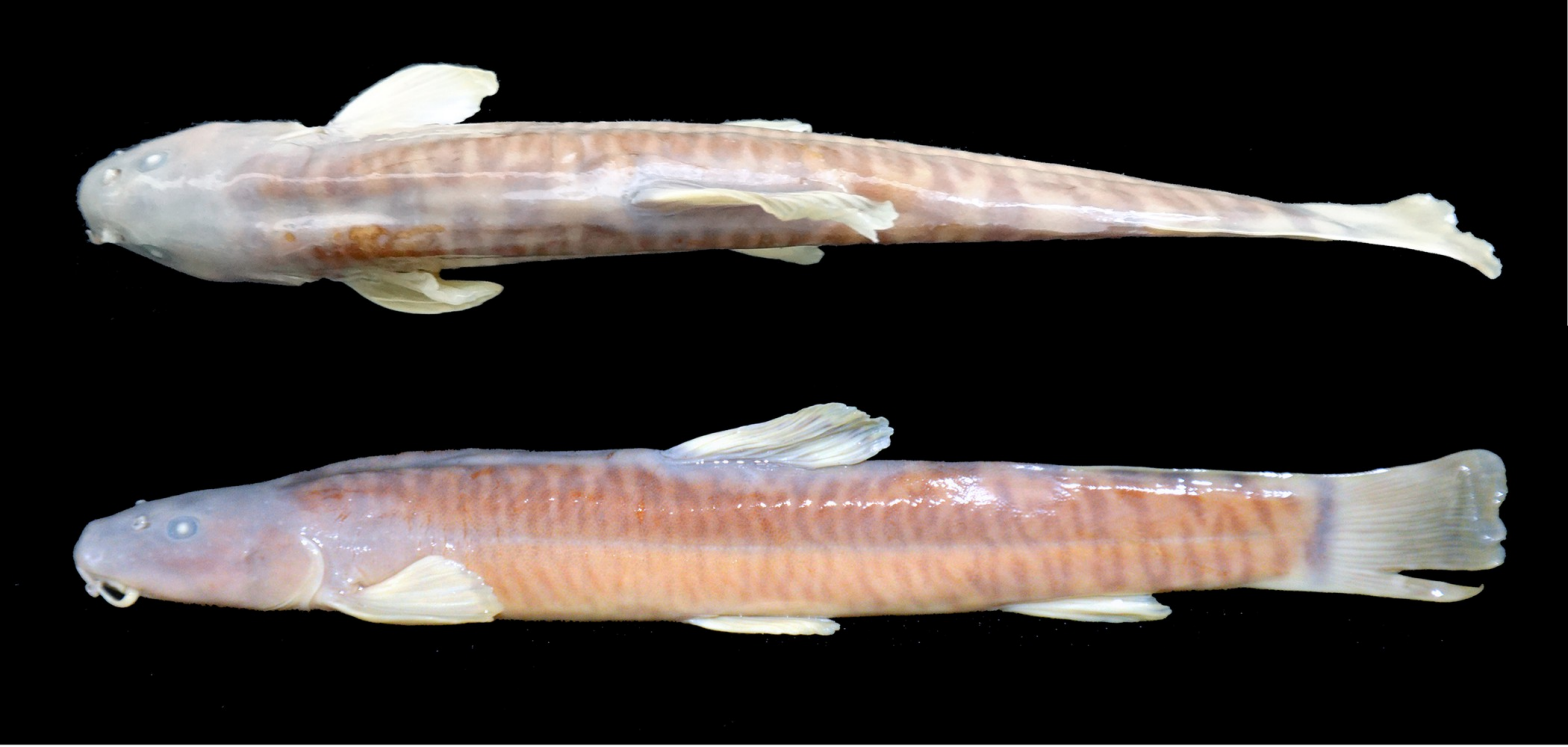
Left lateral and dorsal views of *Homatula microcephala*, holotype SWFU 0612057, 90.2 mm SL; China: Yunnan Prov.: Dali Pref.: Yunlong Co.: Jiancao Town.

LSID: zoobank.org: act: 64A59CCF-60F7-455C-BD1B-DCCB18DF9CB0

*Homatula pycnolepis*: Min et al. 2012a: 81 (Yunnan: Yunlong: Gongguoqiao: Lancang-jiang; Yunnan: Yunlong: Jiuzhou: Lancang-jiang).

**Holotype.** SWFU 0612057, 90.2 mm SL; China: Yunnan Prov.: Dali Pref.: Yunlong Co.: Jiancao Town: Shili-he (26°3’13.58"N, 99°19’17.94"E); collected by Q. Fu, F.L. Li & B. Bai, 20 Dec. 2006.

**Paratypes.** SWFU 0612001–0612017, 0612056, 0612058–0612070, 31 ex., 51.6–130.6 mm SL; the other data are the same as the holotype. SWFU 0612071–0612075, 5 ex., 99.39–110.31 mm SL; China: Yunnan Prov.: Dali Pref.: Yunlong Co.: Baishi Town: Yunding Village (26°17′53.95″N; 99°28′30.15″); collected by Q. Fu, F.L. Li & B. Bai, 20 Dec. 2006.

**Other examined specimens.** KIZ 200410101001 (1 ex., 123.2 mm SL); China: Yunnan Prov.: Dali Pref.: Yunlong Co.: Jiuzhou Town: Gongguoqiao; KIZ 200504272589 (1 ex., 117.4 mm SL); China: Yunnan Prov.: Dali Pref.: Yunlong Co.: Jiuzhou Town: Xiawu Village; KIZ 200504302599–04 (6 ex., 91.7–125.4 mm SL); China: Yunnan Prov.: Dali Pref.: Yunlong Co.: Miaowei Town; KIZ 1974001296, 1974001298–99, 1974001302–03, 1974001307 (6 ex., 104.3–145.2 mm SL); China: Yunnan Prov.: Baoshan City: Longyang District.: Wayao Town: Wayao-he.

**Diagnosis.** *Homatula microcephala* differs from congeners with a densely-scaled body by the following combination of character states: lower jaw spoon-shaped without a median notch (Fig 2C) (*v*. with a marked V-shaped median notch in *H*. *acuticephala*, *H*. *anguillioides*, *H*. *anteriordorsalis*, *H*. *change*, *H*. *coccinocola*, *H*. *cryptoclathratus*, *H*. *niger*, and *H*. *pycnolepis*) (Fig 2B and 2D); paired backwards-extended muscular protrusions between pelvic-fin bases (Fig 3C) (*v*. no paired backwards-extended muscular protrusions between pelvic-fin bases in *H*. *acuticephala*, *H*. *anguillioides*, *H*. *anteriordorsalis*, *H*. *change*, *H*. *coccinocola*, *H*. *cryptoclathratus*, *H*. *erhaiensis*, *H*. *geminusclathratus* sp. nov., *H*. *niger*, and *H*. *wuliangensis*) (Fig 3A); head smaller, its depth 43.9% (39.0–49.0%) HL (*v*. head larger, its depth 54.57% (49.5–61.3%) HL in *H*. *acuticephala*, *H*. *anguillioides*, *H*. *change*, *H*. *coccinocola*, *H*. *erhaiensis*, *H*. *geminusclathratus* sp. nov., *H*. *longibarbatus* sp. nov., *H*. *pycnolepis*, and *H*. *wuliangensis*) (Tables 2 and 3).

**Description.** Based on the holotype and 30 paratypes, the maximum SL is 130.6 mm. Morphometric data are shown in Table 3. Dorsal-fin rays iii, 8_1/2_; anal-fin rays iii, 5_1/2_; pectoral-fin rays i, 9–10; pelvic-fin rays i, 7; caudal-fin branched rays 9+8; vertebra 4+41 (n = 2); 4+42 (n = 1).

Body elongate and cylindrical, anteriorly slightly depressed and posteriorly compressed laterally. Maximum body depth immediately in front of dorsal fin. Dorsal and ventral profiles almost straight. Excepting the head and thorax, body entirely covered by small scales, with scattered small scales on abdomen. Caudal peduncle compressed laterally. Lateral line complete, extending directly along midline of body.

Head short and depressed in frontal view, wider than high, roughly triangular in dorsal view. Snout blunt, slightly shorter than postorbital head. Eye small, close to dorsal profile of head, not visible from ventral view. Interorbital space wide and flat. Nostrils closely set, nearer to anterior margin of eye than to snout tip; anterior nostrils situated at a nostril valve.

Mouth inferior and arched (Fig 2C). Lips thick, slightly furrowed but not papillated; upper lip with a small median incision, and lower lip with a marked median incision. Jaws covered by lips; upper jaw with a well-developed *processus dentiformis*; lower jaw spoon shaped and lacking a notch (Fig 2C). Three pairs of barbels: two rostral pairs, inner pair not reaching corners of mouth and outer pair reaching a vertical line of anterior nostril; one short maxillary pair, reaching vertically to posterior margin of the eye.

Dorsal fin distal margin convex as a whole; dorsal and pelvic origins slightly before midway between snout tip to caudal-fin base. Pectoral fin inserted slightly posterior to vertical through posteriormost point of operculum; tip of adpressed fin rays reaching halfway to insertion of pelvic fin. Pelvic fin inserted below first or second branched rays of dorsal fin, tip of adpressed fin ray approaching halfway between pelvic-fin insertion and anal-fin origin. Pelvic axillary lobe with pointed tip. Anal fin with convex distal edge; origin closer to pelvic-fin insertion than to caudal-fin base. Posterior margin of caudal fin oblique or micro-concave. Caudal peduncle uniformly deep, with adipose crests along its dorsal and ventral midlines. Adipose crest along dorsal midline of caudal peduncle anteriorly not extending vertically through the middle of the anal-fin base (Fig 4B). Anus located approximately posterior, 1/3 the distance between the pelvic-fin insertion and anal-fin origin, nearer to the caudal-fin base.

**Coloration:** In living specimens, the flank brown and the abdomen grayish yellow. The flank has 26 to 30 vertical brownish black bars, some of them joined at the back in a saddle shape. The width of the bar equal to or smaller than the interspace. The bars in front of the dorsal-fin base fine and dense, gradually widening to the caudal-fin base, with a dark brown vertical bar on caudal-fin base. Dorsal, pectoral, pelvic, and anal fins reddish orange, their distal margin orange-red, and fin rays brown. Caudal fin yellowish, distal margin orange-red, and rays brown. After fixation in 95% alcohol, the brown color of the flank and brown-black bars faded; abdomen grayish yellow and orange-red color disappeared on distal edge of each fin.

**Distribution:** Known only from the upper Bi-jiang, a first-class tributary of the Lancang-jiang (Fig 5).

**Habitat and Ecology:** *Homatula microcephala* is found in flowing, clear streams, dwelling on the bottom; loaches travel back and forth among rocks and gravel. It is omnivorous, mainly feeding on algae attached to rocks, organic residues, and some small aquatic insects such as Ephemeroptera. *Homatula microcephala* usually coexists with *Schistura* spp. (Nemacheilidae), *Vanmanenia* spp. (Balitoridae), *Pareuchiloglanis* spp. (Sisoridae), and *Glyptothorax* spp. (Sisoridae).

**Etymology:** From the Latin *micro*–(small) and–*cephala* (a suffix meaning head), indicating that the head of this species is smaller than the congeners of *Homatula*. The name is used as an adjective.

### 3.2 A key to the species of the densely-scaled group of *Homatula*

At present, 13 species of the densely-scaled group of *Homatula* have been recorded in a narrow geographical range from the upper Black River to the Nu-jiang basin. A key to distinguish these species is as follows:

1 No marks on the flank (upper Salween River) *H*. *niger* Li, Che & Zhou

– Bars on the flank vertical along the lateral line 2

2 Bars on the flank across the lateral line no more than 20 3

– Bars on the flank across the lateral line no fewer than 25 5

3 Lower jaw spoon shaped and lacking a notch (upper Mekong River)

*H*. *erhaiensis* (Zhu & Cao)

– Lower jaw with a V-shaped median notch 4

4 Pelvic axillary lobe present (upper Black River)

*H*. *coccinocola* Endruweit, Min & Yang

– Pelvic axillary lobe absent (upper Mekong River) *H*. *acuticephala* (Zhou & He)

5 Adipose crest along mid-dorsal line of caudal peduncle anteriorly reaching through anal-fin origin (upper Black River) *H*. *geminusclathratus* sp. nov.

– Adipose crest along mid-dorsal line of caudal peduncle anteriorly not reaching vertically through middle point of anal-fin base 6

6 Bars on the anterior flank not equally spaced, a few bars occasionally extending from the back down through the lateral line but not reaching the abdomen; bars on the anterior flank usually not obvious in live specimens (upper Salween River)

*H*. *cryptoclathratus* Li, Che & Zhou

– Bars on the anterior flank equally spaced, extending from the back down through the lateral line, reaching the abdomen; bars on the anterior flank clearly visible in live and fixed individuals 7

7 Lower jaw with a V-shaped median notch 8

– Lower jaw spoon shaped and lacking a notch 11

8 Pelvic axillary lobe absent (upper Mekong River) *H*. *anguillioides* (Zhu & Wang)

– Pelvic axillary lobe present 9

9 Adipose crest along mid-dorsal line of caudal peduncle anteriorly extending through end of anal-fin base (upper Mekong River) *H*. *pycnolepis* Hu & Zhang

– Adipose crest along mid-dorsal line of caudal peduncle anteriorly not reaching through end of anal-fin base 10

10 Maxillary barbel almost reaching vertically below middle of the eye (upper Salween River) *H*. *anteriordorsalis* Li, Che & Zhou

– Maxillary barbel extending vertically beyond posterior edge of the eye (upper Black River) *H*. *change* Endruweit

11 Lacking paired backwards-extended muscular protrusions between pelvic-fin bases (upper Mekong River) *H*. *wuliangensis* Min, Yang & Chen

– Paired backwards-extended muscular protrusions between pelvic-fin bases 12

12 Anus located approximately at the last 1/3 between the distance of pelvic-fin insertion and anal-fin origin (upper Mekong River)

*H*. *microcephala* sp. nov.

– Anus located approximately at the last 1/4 between the distance of pelvic-fin insertion and anal-fin origin (upper Mekong River)

*H*. *longibarbatus* sp. nov.

### 3.3 Phylogenetic comparisons among species of *Homatula*

Sequences consisted of 580 conservative sites, 486 variant sites, and 452 reduced information sites. The mean base composition of the sequence was A = 27.6%, T = 29.7%, C = 27.3%, G = 15.4%, A+T = 57.3%, and G+C = 42.7%. The sequences were shown to have little substitution saturation, with a substitution saturation index (Iss) of 0.180, an Iss.S of 0.823, and an Iss.A of 0.791; therefore, the Cyt *b* sequences are not saturated and can be used for phylogenetic inferences. A total of 50 Cyt *b* gene sequences were obtained, and the average sequence length was 1,066 bp.

**Fig 8:**
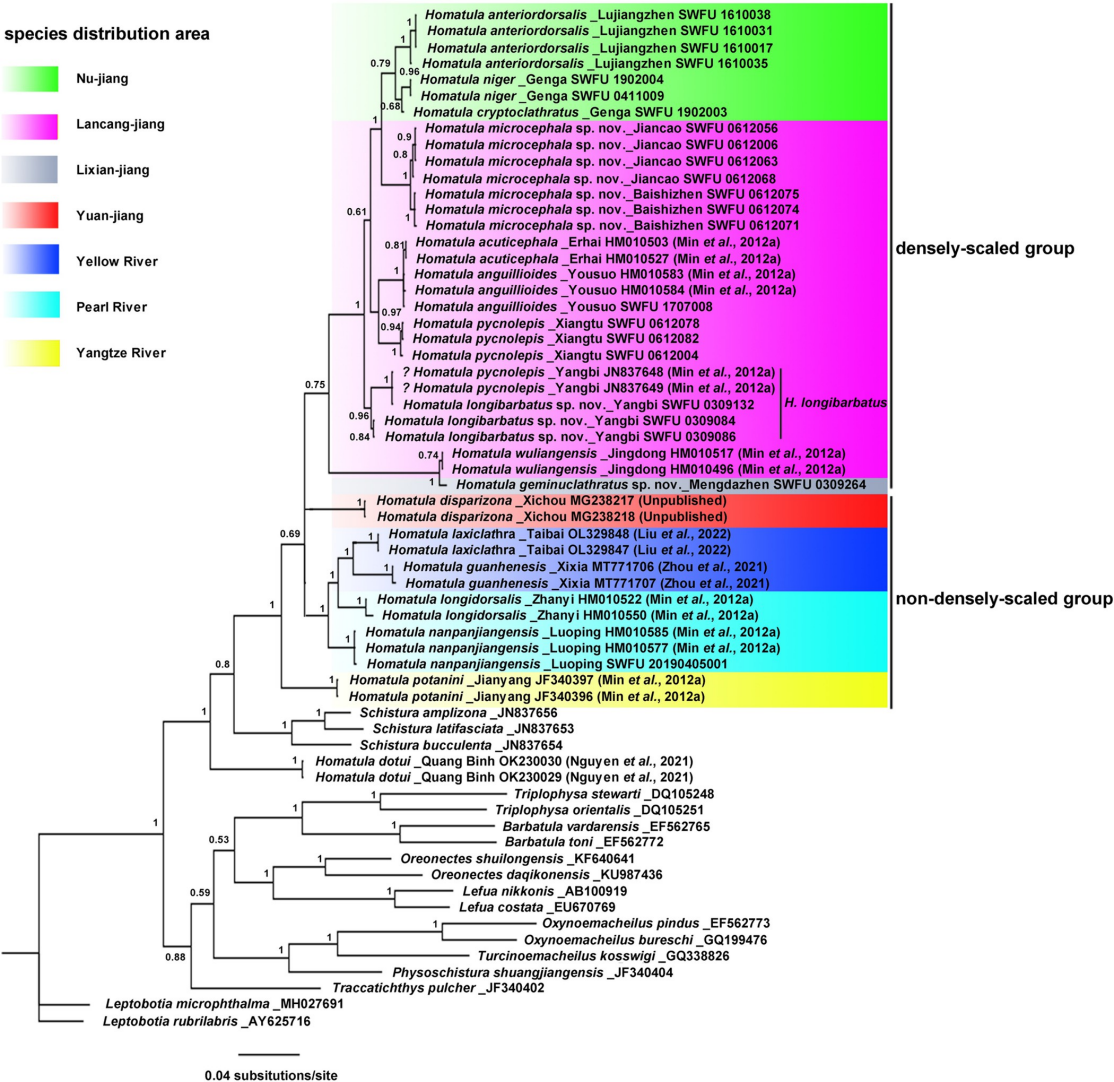
Bayesian phylogeny of *Homatula* inferred from Cyt *b* sequences. Numbers show the posterior probability (PP) values.

In the phylogenetic tree (Fig 8), *H*. *dotui* is sister to a larger clade consisting of all other included *Homatula* species and three *Schistura* species. Aside from *H*. *dotui*, all other *Homatula* species form a monophyletic clade. The densely-scaled group of *Homatula* includes 10 *Homatula* species occurring in the Nu-jiang, the Lancang-jiang, and the upper Black River; this group is monophyletic. The species of the non-densely-scaled group do not form a monophyletic clade. The species of the non-densely-scaled group are clustered into four sub-clades that are constrained to the four river basins: the Red, Pearl, Yangtze, and Yellow River basins.

The three new species, *H*. *microcephala*, *H*. *geminuclathratus*, and *H*. *longibarbatus*, are located in different clades of the phylogeny and are distantly related (Fig 8). *Homatula microcephala* is sister to the *Homatula* species in the Nu-jiang. Moreover, *H*. *geminuclathratus* forms a clade with *H*. *wuliangensis*, and this clade is sister to the rest of the densely-scaled group. It should be noted that two specimens identified by Min et al. [10] as *H*. *pycnolepis* were incorrectly identified. The present study identifies these specimens as *H*. *longibarbatus* (Table 2, Yangbi, Dali, Yunnan; KIZ20100201–202, JN837648–649) both via morphology and molecular phylogenetics because they are not in the same clade with the true *H*. *pycnolepis*, but instead with the new species *H*. *longibarbatus* (Fig 8).

## 4 Discussion

### 4.1 *Homatula* species between the upper Black and the upper Salween Rivers

Evidence from morphological, molecular, and geographical distribution analyses support that the loaches of *Homatula* can be divided into the densely-scaled and non-densely-scaled groups, except for *H*. *dotui*. The species of the densely-scaled group are only distributed in the upper Black River and the upper Nu-jiang. In addition, they share common morphological characteristics (see Tables 1 and 3). Although not all species recorded in the Black River were included in this study, the molecular tree combined with the findings presented in Min et al. [10] (Figs 2–4) and Endruweit et al. [6] (Fig 4) supported the formation of two independent clades of densely-scaled and non-densely-scaled species of *Homatula*. The non-densely scaled group is distributed in the Pearl, Yangtze, and Yellow Rivers, indicating a distinct distribution area that does not overlap with the range of the densely-scaled group (Figs 5 and 8).

The loaches of *Homatula* are widely distributed in the Lancang-jiang and Nu-jiang drainages and had previously been identified as one species, *H*. *pycnolepis* [10, 22]. In this study, the loaches of *Homatula* from these drainages were examined and morphological, molecular, and distribution data support that they should be considered separate species. The morphological findings of this study showed that a paired backwards-extending muscular protrusion between pelvic-fin bases is present in the topotypes and holotype of *H*. *pycnolepis* (Fig 3D). This is a very evident, valuable character that can be used to distinguish *H*. *pycnolepis* from other *Homatula* species. According to this diagnosis, Li, Che & Zhou [4] confirmed that the *H*. *pycnolepis* specimens recorded from the Nu-jiang were misidentified as *H*. *pycnolepis*. In addition, *H*. *pycnolepis*, *H*. *longibarbatus*, and *H*. *microcephala* all have a paired backwards-extending muscular protrusion between pelvic-fin bases, yet other morphological characters are distinct (Fig 3B–3D). Molecular phylogenetics supported the naming of three new species: *H*. *geminusclathratus* sp. nov., *H*. *microcephala* sp. nov., and *H*. *longibarbatus* sp. nov. (Fig 8). The species of *Homatula* distributed in the Lancang-jiang and the Chuan-he (upper Black River) have distinct distributions in different regions. *Homatula pycnolepis* and *H*. *longibarbatus* are distributed in the Yangbi-jiang (the upper Heihui-jiang, a first-class tributary of the Lancang-jiang), and *H*. *microcephala* occurs in the Bi-jiang (a first-class tributary of the Lancang-jiang). *H*. *geminusclathratus* is distributed on the eastern slope of Wuliangshan, which flows into the Chuan-he basin (Fig 5). Although these three new species are distributed in different tributaries of the Lancang-jiang and Lixiang-jiang, the distribution area of these three new species is very close geographically.

Previous studies have found conflicting results about the status and validity of *Homatula anguillioides*, *H*. *acuticephala*, *H*. *erhaiensis*, and *H*. *pycnolepis* [2, 6, 10]. Specifically, Min et al. [10] suggested that *H*. *acuticephala* was a synonym of *H*. *anguillioides*. However, based on morphological characters, Hu & Zhang [2] supplied convincing evidence that these four species were valid. The molecular tree presented in this study supports *H*. *acuticephala* as a synonym of *H*. *anguillioides*, and *H*. *pycnolepis* as a separate species. In the absence of molecular evidence for *H*. *erhaiensis*, it would be arbitrary to treat it as a synonym for *H*. *acuticephala* because its morphology is markedly different from the other three species. Future molecular studies should be conducted on *H*. *erhaiensis* to clarify its position as a valid species.

### 4.2 The status of *Homatula dotui*

The results of the phylogenetics analysis suggest that the taxonomic status of *Homatula dotui* is questionable, and it may not be a species in the genus *Homatula* (Fig 8). Other evidence also suggests that *H*. *dotui* may be in a different genus. Specifically, Nguyen et al. [14] failed to fully demonstrate that *H*. *dotui* should belong in the genus *Homatula*. In their study, *H*. *dotui* was distantly related to other species of *Homatula*; thus, *H*. *dotui* was placed in the genus *Homatula*. However, using the molecular data from Nguyen et al. [14], the molecular tree results calculated in this study showed that *H*. *dotui* and other *Homatula* species did not form a monophyletic group, and instead, it intermixed with outgroup species (Fig 8).

Several important morphological characters of the original description and figures of *H*. *dotui* [14] were inconsistent with the characters used to distinguish *Homatula* from its relatives in the Nemacheilidae [2, 23, 24], namely, caudal fin slightly concave or deeply concave (vs. truncated or convex); dorsal of the frontal distinctly raised from lateral view (vs. flat); fewer vertebrae, 4+31 (vs. more than 4+33). In addition, neither the original description nor the figures illustrated the morphological characters of the lips and jaws [14], which are indispensable for distinguishing *Homatula* from similar genera of the Nemacheilidae.

The distribution of *H*. *dotui* is discontinuous with that of other species of *Homatula*. The type locality of *H*. *dotui* is in Quang Binh Province, Vietnam, and the specimens were collected from a cave that connected to the Gianh River, which flows directly into the Gulf of Tonkin [14]. Until *H*. *dotui* was described, there were no records of *Homatula* in Vietnam. The locality of *Homatula change* in the upper Black River of Yunnan, China is the closest *Homatula* species to the *H*. *dotui* collection site. However, these locations are very distant and belong to different river basins [5]. No *Homatula* has been recorded in the area between the Black River basin and the Gianh River. Therefore, molecular, morphological, and distribution data suggest that *H*. *dotui* should be carefully re-examined. Future studies should be conducted to resolve the classification of *H*. *dotui*.

### 4.3 Conservation of *Homatula* habitat

The species of the densely-scaled group of *Homatula* are strictly distributed in the area between the Lixian-jiang and Nu-jiang basins [4]. No specimens of *Homatula* were collected from the main stream of the Lixian-jiang, Lancang-jiang, and Nu-jiang nor their large tributaries. The loaches of *Homatula* only inhabit mountain streams with rapid or gentle currents, vauclusian springs, underground rivers connected to streams, and ditches near villages and farmland [2, 5–8, 24–28]. The water in their habitat is clear and pollution-free. The water depth is generally 30–50 cm, and the bottom is mostly rocks, gravel, and sand, with algae on the surface of rocks and gravel, and a small amount of water grass growing on the bottom. There is typically only one companion fish species and it may be from *Schistura*, *Devario*, *Schizothorax*, or Sisoridae catfish of *Creteuchiloglanis*, *Glyptothorax*, and *Pareuchiloglanis*.

Because the loaches of *Homatula* are weak swimmers and are benthic, most species of *Homatula* are restricted to a narrow region, which may have led to geographical isolation, accelerating their morphological differentiation. *Homatula* species usually exist in small populations; thus, they are vulnerable to extinction. Small environmental changes in their habitat, such as water pollution or extensive human use, can lead to species or population extinction. For example, *Homatula erhaiensis* lived in the streams that flow into the eastern side of Erhai Lake, Dali City. Due to drought, these streams have dried up, and *H*. *erhaiensis* has not been recorded in the eastern shore of Erhai Lake or its surrounding areas since the 1980s. In addition, the type specimen of *H*. *anguillioides* is from Eryuan County, yet Dr. Zhou, one of the authors, was unable to find this species until July 2017, when it was found in a vauclusian spring with *Schizothorax taliensis*. This vauclusian spring is connected to underground caves and can be protected as a fish resource reserve, which would allow for the protection of *H*. *anguillioides* and *S*. *taliensis*. *Homatula geminusclathratus* was previously found in a stream next to Changdi Village, Wenjing Town, Jingdong County in 2010 and 2011. Due to human activity and intensive collection, almost no specimens can currently be found. *Homatula microcephala* lives in the tributaries of Bijiang, Yunlong County, where it is difficult to find due to the water pollution caused by upstream mining.

To conserve the loaches of *Homatula*, it is urgent to construct small protection areas. At present, some springs and vauclusian springs where the loaches of *Homatula* can be found are water sources or religious sites, and these areas are mostly protected. Conversely, other areas inhabited by loaches of *Homatula* are impacted by human activity, particularly at river headwaters and in areas with currents. It may not be appropriate to establish a large reserve to protect rare, endemic fishes that are scattered throughout a river basin. For rare, endemic fishes, like the loaches of *Homatula*, targeted protection rather than an extensive protection area, is needed.

## Supporting information

S1 File (DOCX)

S1 Appendix Species used in the molecular study [29–33]. (DOCX)
