# Supplementary material for: Three new species of Homatula (Teleostei: Nemacheilidae) from Yunnan, China, with comments on habitat conservation
Source: PLoS One. 2022 Nov 23;17(11):e0276846. doi: 10.1371/journal.pone.0276846 (PMC9683601; doi:10.1371/journal.pone.0276846)
Supplement: S1 File — (DOCX) [file pone.0276846.s001.docx]

### MATERIAL EXAMINED

*Homatula* *acuticephala*: KIZ 784141 (1 ex., holotype, 109.5 mm *L*_S_); KIZ 2008005990–6015 (26 ex., 33.7–51.5 mm *L*_S_); Haixi-hai, Niujie Town, Eryuan Co., Dali Pref., Yunnan Prov., China.

*Homatula anguillioides*: KIZ 2008006532–43 (12 ex., 68.8–143.3 mm *L*_S_); Yousuo Spring, Eryuan Co., Dali Pref., Yunnan Prov., China; SWFU 1707001–1707009 (9 ex., 75.7–151.5 mm *L*_S_); a spring, Yousuo Town, Eryuan Co., Dali Pref., Yunnan Prov., China.

*Homatula anteridorsalis*: SWFU 1610006 (1 ex., holotype, 121.6 mm *L*_S_); SWFU 1610001-1610005, 1610007-1610031 (paratypes, 30 ex., 66.4–129.4 mm *L*_S_); Bingmen Village, Lujiang Town, Longyang District, Baoshan City, Yunnan Prov., China; SWFU 1510001-1510002 (2 ex., 108.0–140.8 mm *L*_S_); Kungong Village, Lujiang Town, Longyang District, Baoshan City, Yunnan Prov., China.

*Homatula berezowskii*: SWFU 1807001–1807020 (20 ex., 60.5–137.4 mm *L*_S_); Miyuesi, Feng Co., Baoji City, Shaanxi Prov., China.

*Homatula change*: KIZ 2012004205 (1 ex., holotype, 107.6 mm *L*_S_); KIZ 2012004208, 4209, 4211, 4215–18, 4221–24 (11 ex., paratypes, 37.9–76.5 mm *L*_S_); a tributary to Mengye-jiang, hill stream close to Yiwanshui Village, Jiangcheng Co., Pu’er City, Yunnan Prov., China; SWFU 0412001–0412006, 0512001–0512002 (8 ex., 105.7–150.5 mm *L*_S_); Jima-he, Jiangcheng Co., Pu'er City, Yunnan Prov., China.

*Homatula coccinocola*: KIZ 2011002847 (1 ex., holotype, 99.6 mm *L*_S_); KIZ 2012001866–1869 (4 ex., paratypes, 51.1–79.0 mm *L*_S_); Tengtiao-jiang (a branch of the Red River), Jiache Town, Honghe Co., Honghe Pref., Yunnan Prov., China.

*Homatula cryptoclathrata*: SWFU 1002001 (1 ex., holotype, 138.1 mm *L*_S_); SWFU 1902002-19020011 (10 ex., paratypes, 53.2-110.1 mm *L*_S_); Manping Village, Gengga Town, Changning Co., Baoshan City., Yunnan Prov., China; KIZ 2006009298, 2006009312, 2006009315, 2006009317–21, 2006009389–92, 2006009398–99 (14 ex., 77.5–119.8 mm *L*_S_); Gongyang-he, Longling Town, Baoshan City., Yunnan Prov., China.

*Homatula disparizona*: KIZ 2012000623 (1 ex., holotype, 76.0 mm *L*_S_); KIZ 2012000622, 0624–0634 (12 ex., paratypes, 44.3–78.1 mm *L*_S_); KIZ 2012001859–1865 (7 ex., 58.4–72.8 mm *L*_S_); Panlong-he, Xichou Co., Wenshan Pref., Yunnan Prov., China.

*Homatula dotui* data from (Nguyen et al., 2021) [14].

*Homatula* *erhaiensis*: IHB 64VI0012 (1 ex., holotype, 68.8 mm *L*_S_); IHB 64VI0001–11, 64VI0013–5, 646775–7, 646779 (18 ex., paratypes, 64.4–86.8 mm *L*_S_); IHB 1270142–8, 1270150–4 (12 ex., 49.1–79.9 mm *L*_S_); Wase Town on the eastern shore of Erhai Lake (upper Mekong River drainage), Dali Pref., Yunnan Prov., China.

*Homatula guanheensis* data from (Zhou et al., 2021) [13].

*Homatula laxiclathra*: data from (Gu & Zhang, 2012) [26].

*Homatula longidorsalis*: KIZ 874042–874043, 874045–874047, 874050, 874195–874216 (28 ex., paratypes, 46.1–89.5 mm *L*_S_); SWFU 0403001–0403027 (27 ex., 57.5–87.4 mm *L*_S_); Jiuxiang Town, Yiliang Co., Kunming City, Yunnan Prov., China.

*Homatula nanpanjiangensis*: KIZ 1994000023 (1 ex., holotype, 86.4 mm *L*_S_); KIZ 19940018–19940022, 19940024–19940037 (19 ex., paratypes 64.7–89.4 mm *L*_S_); Niujie River (a tributary of Nanpan-jiang at Niujie Town), Luoping Co., Qujing City, Yunnan Prov., China.

*Homatula nigra*: SWFU 1901005 (1 ex., holotype, 99.8 mm *L*_S_); SWFU 1901001-1901004 (4 ex., 92.0–109.1 mm *L*_S_); SWFU 0411005-0411009 (5 ex., 55.2–87.4 mm *L*_S_); Xiangshui Village, Gengga Town, Changning Co., Baoshan City, Yunnan Prov., China.

*Homatula oligolepis*: KIZ 774557–560 (4 ex., 75.5–114.2 mm *L*_S_); KIZ 19850829–19850830 (2 ex., 127.8–169.7 mm *L*_S_); Zhanyi Co., Qujing City, Yunnan Prov., China.

*Homatula oxygnathra* data from (Liu, Cao & Zhang, 2022) [15].

*Homatula potanini*: KIZ 2013004619–2013004621 (3 ex., 53.8–66.2 mm *L*_S_); Gaojiayan Town, Changyang Co., Yichang City, Hubei Prov., China.

*Homatula pycnolepis*: IHB 814045 (1 ex., holotype, 118.8 mm *L*_S_); Shaxi Town, Jianchuan Co., Dali Pref., Yunnan Prov., China; SWFU 0612076–0612090 (15 ex., 61.8–137.2 mm *L*_S_); Xiangtu Town, Yunlong Co., Dali Pref., Yunnan Prov., China; KIZ 2009005388, 2010002517–2554 (39 ex., 83.2–145.5 mm *L*_S_); Yangbi Co., Dali Pref., Yunnan Prov., China.

*Homatula robusta* data from (Min et al., 2022) [27].

*Homatula variegata*: SWFU 1810007–1810033 (27 ex., 43.7–100.7 mm *L*_S_); Wangjia-he, Zhouzhi Co., Xian City, Shaanxi Prov., China.

*Homatula wenshanensis*: SWFU 0201037–0201042, 0201044–0201046 (9 ex., 67.7–90.0 mm *L*_S_); Shuidian-he, Wenshan Co., Wenshan Pref., Yunnan Prov., China.

*Homatula wujiangensis*: data from (Ding & Deng, 1990) [28].

*Homatula wuliangensis*: KIZ 2008008158 (1 ex., holotype, 181.9 mm *L*_S_); KIZ 2008008156–157, 159–172, 175–176, 179, 184, 197, 199–201, 203, 205, 207, 211, 214–215, 316–318 (33 ex., paratypes, 64.6–191.1 mm *L*_S_); Baimushan-he, Jingfu Town, Jingdong Co., Pu’er City, Yunnan Prov., China; SWFU 9804001–9804019 (19 ex., 72.4–178.0 mm *L*_S_); Jingfu Town, Jingdong Co., Pu’er City, Yunnan Prov., China.
