# Supplementary material for: Three new species of Homatula (Teleostei: Nemacheilidae) from Yunnan, China, with comments on habitat conservation
Source: PLoS One. 2022 Nov 23;17(11):e0276846. doi: 10.1371/journal.pone.0276846 (PMC9683601; doi:10.1371/journal.pone.0276846)
Supplement: S1 Appendix — (DOCX) [file pone.0276846.s002.docx]

**Appendix 1.** Species used in the molecular study

| Species | Collection locality | Voucher No. | GenBank Accession No. | Data source |
| --- | --- | --- | --- | --- |
| Outgroup |  |  |  |  |
| *Barbatula toni* | Korea | Unknow | EF562772 | [29] Šedivá et al. (2008) |
| *Barbatula vardarensis* | Macedonia | Unknow | EF562765 | [29] Šedivá et al. (2008) |
| *Lefua costata* | Amur River, Korea | Unknow | DQ105196 | Unpublished |
| *Lefua nikkonis* | Ono River, Japan | Unknow | AB100919 | [30] Saka et al. (2003) |
| *Leptobotia microphthalma* | Min River, China | Unknow | MH027691 | Unpublished |
| *Leptobotia rubrilabris* | Yangtze River, China | IHCAS0000021 | AY625716 | [31] Tang et al. (2006) |
| *Oreonectes daqikongensis* | Liu River, China | Unknow | KU987436 | Unpublished |
| *Oreonectes shuilongensis* | Liu River, China | Unknow | KF640641 | [32] Deng et al. (2016) |
| *Oxynoemacheilus bureschi* | Balkan Peninsula | Unknow | GQ199476 | [33] Šedivá et al. (2010) |
| *Oxynoemacheilus pindus* | Albania | Unknow | EF562773 | [33] Šedivá et al. (2010) |
| *Physoschistura shuangjiangensis* | Lancang River, China | KIZ20050407015 | JF340404 | [10] Min et al. (2012a) |
| *Schistura amplizona* | Yunnan, China | KIZ2010003103 | JN837656 | [10] Min et al. (2012a) |
| *Schistura bucculenta* | Yunnan, China | KIZ20080614 | JN837654 | [10] Min et al. (2012a) |
| *Schistura latifasciata* | Yunnan, China | KIZ20050325004 | JN837653 | [10] Min et al. (2012a) |
| *Traccatichthys pulcher* | China | KIZ20051017041 | JF340402 | [10] Min et al. (2012a) |
| *Triplophysa orientalis* | Yangtze River, China | IHCAS0405365 | DQ105251 | [31] Tang et al. (2006) |
| *Triplophysa stewarti* | Qinghai, China | IHCAS0307103 | DQ105248 | [31] Tang et al. (2006) |
| *Turcinoemacheilus kosswigi* |  | DFK2012562 | KU180213 | Unpublished |
| Ingroup |  |  |  |  |
| Homatula |  |  |  |  |
| *Homatula acuticephala* | Dali, Yunnan | KIZ2008005994 | HM010503 | [10] Min et al. (2012a) |
| *Homatula acuticephala* | Dali, Yunnan | KIZ2008005993 | HM010527 | [10] Min et al. (2012a) |
| *Homatula anguillioides* | Eryuan, Dali, Yunnan | KIZ20080304 | HM010583 | [10] Min et al. (2012a) |
| *Homatula anguillioides* | Eryuan, Dali, Yunnan | KIZ20080306 | HM010584 | [10] Min et al. (2012a) |
| *Homatula anguillioides* | Eryuan, Dali, Yunnan | SWFU1707008 |  | this study |
| *Homatula anteriordorsalis* | Bingmen, Lujiangzhen, Baoshan, Yunnan | SWFU1610038 |  | this study |
| *Homatula anteriordorsalis* | Bingmen, Lujiangzhen, Baoshan, Yunnan | SWFU1610031 |  | this study |
| *Homatula anteriordorsalis* | Bingmen, Lujiangzhen, Baoshan, Yunnan | SWFU1610035 |  | this study |
| *Homatula anteriordorsalis* | Bingmen, Lujiangzhen, Baoshan, Yunnan | SWFU1610017 |  | this study |
| *Homatula cryptoclathratus* | Gengga, Changning, Baoshan, Yunnan | SWFU1902003 |  | this study |
| *Homatula disparizona* | Xichou, Wenshan, Yunnan | KIZ2012000622 | MG238217 | Unpublished |
| *Homatula disparizona* | Xichou, Wenshan, Yunnan | KIZ2012000626 | MG238218 | Unpublished |
| *Homatula dotui* | Vietnam | IEBR00439 | OK230030 | [14] Nguyen et al. (2021) |
| *Homatula dotui* | Vietnam | IEBR00440 | OK230029 | [14] Nguyen et al. (2021) |
| *Homatula geminuclathratus* | Jingdong, Yunnan | SWFU0309264 |  | this study |
| *Homatula guanhensis* | Guanhe, Xixia, Henan | HUN101134 | MT771706 | [13] Zhou et al. (2021) |
| *Homatula guanhensis* | Guanhe, Xixia, Henan | HUN101135 | MT771707 | [13] Zhou et al. (2021) |
| *Homatula laxiclathra* | Shitou-He, Taibai, Shaanxi | IHB202106056010 | OL329847 | [15] Liu, Cao & Zhang (2022) |
| *Homatula laxiclathra* | Shitou-He, Taibai, Shaanxi | IHB202106056009 | OL329848 | [15] Liu, Cao & Zhang (2022) |
| *Homatula longibarbatus* | Yuejinxiang, Yangbi, Dali, Yunnan | SWFU0309132 |  | this study |
| *Homatula longibarbatus* | Pingpo, Yangbi, Dali, Yunnan | SWFU0309084 |  | this study |
| *Homatula longibarbatus* | Pingpo, Yangbi, Dali, Yunnan | SWFU0309086 |  | this study |
| *Homatula longidorsalis* | Zhanyi, Yunnan | KIZ20060274 | HM010522 | [10] Min et al. (2012a) |
| *Homatula longidorsalis* | Zhanyi, Yunnan | KIZ20060276 | HM010550 | [10] Min et al. (2012a) |
| *Homatula microcephala* | Baishi, Yunlong, Dali, Yunnan | SWFU0612075 |  | this study |
| *Homatula microcephala* | Baishi, Yunlong, Dali, Yunnan | SWFU0612074 |  | this study |
| *Homatula microcephala* | Baishi, Yunlong, Dali, Yunnan | SWFU0612071 |  | this study |
| *Homatula microcephala* | Jiancao, Yunlong, Dali, Yunnan | SWFU0612056 |  | this study |
| *Homatula microcephala* | Jiancao, Yunlong, Dali, Yunnan | SWFU0612006 |  | this study |
| *Homatula microcephala* | Laidengwei, Jiancao, Yunlong, Dali, Yunnan | SWFU0612063 |  | this study |
| *Homatula microcephala* | Laidengwei, Jiancao, Yunlong, Dali, Yunnan | SWFU0612068 |  | this study |
| *Homatula nanpanjiangensis* | Luoping, Yunnan | KIZ20080433 | HM010585 | Unpublished |
| *Homatula nanpanjiangensis* | Luoping, Yunnan | KIZ20080432 | HM010577 | Unpublished |
| *Homatula nanpanjiangensis* | Luoping, Yunnan | SWFU20190405001 |  | this study |
| *Homatula niger* | Gengga, Changning, Baoshan, Yunnan | SWFU1902004 |  | this study |
| *Homatula niger* | Gengga, Changning, Baoshan, Yunnan | SWFU0411009 |  | this study |
| *Homatula potanini* | Jianyang, Sichuan | KIZ2010000237 | JF340397 | [10] Min et al. (2012a) |
| *Homatula potanini* | Jianyang, Sichuan | KIZ2010000234 | JF340396 | [10] Min et al. (2012a) |
| ? *Homatula pycnolepis* | Yangbi, Dali, Yunnan | KIZ20100201 | JN837648 | [10] Min et al. (2012a) |
| ? *Homatula pycnolepis* | Yangbi, Dali, Yunnan | KIZ20100202 | JN837649 | [10] Min et al. (2012a) |
| *Homatula pycnolepis* | Xiangtuxiang, Yunlong, Dali, Yunnan | SWFU0612078 |  | this study |
| *Homatula pycnolepis* | Xiangtuxiang, Yunlong, Dali, Yunnan | SWFU0612082 |  | this study |
| *Homatula pycnolepis* | Xiangtuxiang, Yunlong, Dali, Yunnan | SWFU0612004 |  | this study |
| *Homatula wuliangensis* | Jingdong, Yunnan | KIZ20080581 | HM010517 | [10] Min et al. (2012a) |
| *Homatula wuliangensis* | Jingdong, Yunnan | KIZ20080582 | HM010496 | [10] Min et al. (2012a) |
